# Rationale, Design, and Participant Baseline Characteristics of a Parallel Randomized Trial of the Effect of Replacing SSBs with Cow’s Milk Versus Soymilk on Intrahepatocellular Lipid and Other Cardiometabolic Risk Factors in Adults with Obesity Who Consume Sugar-Sweetened Beverages: The Soy Treatment Evaluation for Metabolic health (STEM) Trial

**DOI:** 10.3390/nu18071026

**Published:** 2026-03-24

**Authors:** Madeline N. Erlich, Diana Ghidanac, Sonia Blanco Mejia, Sabrina Ayoub-Charette, Claudia Vittes Combe, Tauseef A. Khan, Devina Ramdath, Heather Crewson, Amanda Beck, Constança Silva, D. Dan Ramdath, Adam H. Metherel, Lawrence A. Leiter, Richard P. Bazinet, Cyril W. C. Kendall, David J. A. Jenkins, Laura Chiavaroli, John L. Sievenpiper

**Affiliations:** 1 Department of Nutritional Sciences, Temerty Faculty of Medicine, University of Toronto, Toronto, ON M5S 1A8, Canada; 2 Toronto 3D Knowledge Synthesis and Clinical Trials Unit, Clinical Nutrition and Risk Factor Modification Centre, St. Michael’s Hospital, Toronto, ON M5B 1W8, Canada; 3 Department of Family Relations and Applied Nutrition, University of Guelph, Guelph, ON N1G 2W1, Canada; 4 Clinical Nutrition Department, Civic Campus, The Ottawa Hospital, Ottawa, ON K1Y 4E9, Canada; 5 Division of Cardiac Prevention and Rehabilitation, University of Ottawa Heart Institute, Ottawa, ON K1Y 4W7, Canada; 6 College of Pharmacy and Nutrition, University of Saskatchewan, Saskatoon, SK S7N 5E5, Canada; 7 Li Ka Shing Knowledge Institute, St. Michael’s Hospital, Toronto, ON M5B 1A6, Canada; 8 Department of Medicine, Temerty Faculty of Medicine, University of Toronto, Toronto, ON M5S 1A8, Canada; 9 Division of Endocrinology and Metabolism, Department of Medicine, St. Michael’s Hospital, Toronto, ON M5B 1W8, Canada

**Keywords:** sugar-sweetened beverages, soymilk, cow’s milk, randomized controlled trial, abdominal obesity, overweight, type 2 diabetes, liver fat, glucose control, insulin sensitivity, metabolic impairment, obesity, dietary intervention

## Abstract

**Background/Objectives**: Liver fat represents an early metabolic lesion in the development of diabetes and its cardiometabolic complications. Diets high in free sugars, particularly from sugar-sweetened beverages (SSBs), are associated with abdominal obesity and increased cardiometabolic risk, prompting global guidelines to limit SSBs as a major public health strategy. Low-fat cow’s milk is promoted as the preferred caloric replacement strategy for SSBs due to its high nutritional value and cardiometabolic advantages. Fortified soymilk is a plant-based alternative with approved health claims for cholesterol and coronary heart disease risk reduction that offers an equivalent nutritional value to cow’s milk. However, given concerns about its classification as an ultra-processed food (UPF), it is unclear whether soymilk offers comparable metabolic health benefits to milk as part of clinical and public health strategies to reduce SSB intake. The Soy Treatment Evaluation for Metabolic (STEM) health trial seeks to evaluate the impact of replacing SSBs with either 2% soymilk or 2% cow’s milk on liver fat and other cardiometabolic risk factors in habitual adult consumers of SSBs with obesity. **Methods**: The STEM trial is a 24-week, pragmatic, 3-arm, parallel, randomized trial. We recruited adults with obesity (high BMI plus high waist circumference based on ethnic specific cut-offs) consuming ≥1 SSB/day. Participants were randomized to one of three groups based on their usual SSB intake at baseline (servings/day): continued SSB (355 mL can) intake; replacement with fortified, sweetened 2% soymilk (250 mL); or replacement with 2% cow’s milk (250 mL). The primary outcome is the change in intrahepatocellular lipid (IHCL) measured by ^1^H-MRS at 24 weeks. Hierarchical testing will be done to reduce the familywise error rate. The superiority of cow’s milk to SSBs will be assessed first to establish assay sensitivity. If superiority is established, then the non-inferiority of soymilk to cow’s milk will be assessed using a pre-specified non-inferiority margin of 1.5% IHCL units (assessed by difference of means using a 90% confidence interval [CI]). Analyses will be conducted according to the intention-to-treat (ITT) principle using inverse probability weighting (IPW) for superiority testing and per-protocol analyses for non-inferiority testing, using ANCOVA adjusted for age, sex, metabolic dysfunction-associated steatotic liver disease (MASLD) status, medication use, intervention dose, and baseline levels. We hypothesize that soymilk will be non-inferior to cow’s milk (Clinicaltrials.gov NCT05191160). **Results**: Recruitment began in November 2021. A total of 3050 individuals were screened. We randomized 186 participants (62 per group) between 19 April 2022 and 16 April 2024. Participants are 57% male; with a mean [SD] age of 39.9 [11.8] years; BMI of 34.6 [6.1] kg/m^2^, waist circumference of 112.6 [13.8] cm; IHCL of 10.0 [8.2] % with 64.1% meeting the criteria for MASLD; and SSBs intake of 2.3 [1.3] servings/day. **Conclusions**: Baseline characteristics were balanced across the study arms, with participants representing adults with a high-risk metabolic phenotype, and 64.1% meeting the criteria for MASLD. Findings will contribute to evidence on the cardiometabolic benefits of soymilk, informing clinical practice guidelines and public health policy.

## 1. Introduction

Abdominal obesity, defined by excess visceral fat accumulation, is a key driver of cardiometabolic risk and is central to the global epidemics of obesity and type 2 diabetes [1,2]. Excess liver fat, in particular, is recognized as an early lesion of metabolic dysfunction and a critical mediator in the progression of cardiometabolic disease, as outlined in the twin cycle hypothesis [3]. The twin cycle hypothesis proposes that chronic excess energy intake drives progressive accumulation of fat in the liver and pancreas, leading to hepatic insulin resistance, impaired glucose regulation, and a self-reinforcing cycle that culminates in the development of type 2 diabetes [3].

In this context, diets high in free sugars have been consistently implicated in the development of liver fat and in contributing substantially to abdominal obesity, prompting global health authorities to recommend limiting free sugars to less than 10% of total energy intake [4,5,6,7]. Among the sources of dietary free sugars, sugar-sweetened beverages (SSBs) are the primary target of public health strategies aimed at reducing sugar intake because they contribute to excess energy intake, weight gain, and increases in cardiometabolic risk, and are associated with obesity, diabetes, and cardiovascular disease [4,8,9,10,11,12,13,14,15]. Although consumption of SSBs has declined in recent years, they remain the leading source of added sugars in the North American diet [8,16]. Water is widely promoted as the ideal non-caloric replacement for SSBs but may not be a practical option in all contexts [17,18]. Low-fat cow’s milk is the recommended caloric replacement in SSB-reduction strategies, as reflected in procurement, vending machine, and menu-planning policies across educational, healthcare, and other institutional settings, due to its association with improved intermediate cardiometabolic outcomes [19,20,21,22,23,24,25,26,27].

However, there is increasing interest in plant-based alternatives to cow’s milk. Major dietary guidelines increasingly support a shift toward more plant-based dietary patterns, motivated by both public health and environmental considerations [17,18,28,29]. This shift has contributed to the proliferation of plant-based dairy alternatives. Fortified soymilk is the only plant-based beverage recognized as nutritionally comparable to cow’s milk by the Dietary Guidelines for Americans (2020–2025), Canada’s Food Guide, and several European dietary guidelines [17,18,30]. As a source of soy protein, it also carries approved health claims from Health Canada, the U.S. Food and Drug Administration (FDA), and 8 other jurisdictions for cholesterol and coronary heart disease risk reduction (summarized in Supplementary Table S1) [31,32,33,34,35,36,37,38,39,40]. Nevertheless, the World Health Organization (WHO)-endorsed NOVA classification system categorizes soymilk as an ultra-processed food (UPF) to be avoided [41]. This classification presents a significant barrier to the broader adoption of soymilk in institutional programs, such as school nutrition initiatives, which have traditionally provided funding to unprocessed cow’s milk [20]. Soymilk is frequently formulated with added sugars to match the natural sweetness of cow’s milk, which disqualifies it from meeting the FDA’s proposed criteria for foods labelled as “healthy” [42]. As the demand for plant-based alternatives rises, there is an increasing need to critically evaluate the role of nutrient-dense foods classified as UPFs, such as soymilk, within public health nutrition programs and policy frameworks. A recent systematic review and meta-analysis of randomized trials evaluated the effects of substituting soymilk for minimally processed cow’s milk on intermediate cardiometabolic outcomes and assessed whether these effects differed by added sugar content [43]. The findings provide a good indication that replacing cow’s milk with soymilk does not adversely affect a range of intermediate cardiometabolic outcomes, regardless of added sugar content, and may offer benefits for blood lipids, blood pressure, and inflammation in adults with varying health statuses. The benefits of substituting soymilk for cow’s milk for LDL and blood pressure reduction were echoed in a 2026 network meta-analysis of plant-based drinks on cardiometabolic outcomes [44]. These results challenge prevailing public health paradigms that position soymilk as a food to be avoided. However, follow-up durations across the existing evidence are limited, with a median of four weeks (range, 4–16 weeks), which may be insufficient to observe changes in outcomes that require a longer intervention period [45]. Existing evidence does not address all relevant intermediate cardiometabolic outcomes, most notably hepatic fat, as a sensitive marker of early metabolic dysfunction. A randomized controlled trial evaluating the effect of substituting cow’s milk for SSBs demonstrated reductions in liver fat at 24 weeks, which investigators suggested may reflect the displacement of rapidly absorbable fructose, a driver of hepatic de novo lipogenesis [45]. No such trial has evaluated the replacement of SSBs with soymilk or directly compared soymilk and cow’s milk on liver fat [45].

The STEM trial aims to investigate whether soymilk is a viable, healthy, plant-based alternative to cow’s milk in SSB-reduction strategies. The trial compares the effects of replacing SSBs with 2% soymilk or 2% cow’s milk on intrahepatocellular lipid (IHCL) and a comprehensive range of cardiometabolic risk factors in habitual SSB consumers at elevated risk for cardiometabolic disease. As a long-term randomized trial that directly compares soymilk and cow’s milk within an SSB-reduction framework, this study will provide critical evidence on the role of soymilk in supporting metabolic health and informing future dietary guidance.

## 2. Materials and Methods

### 2.1. Trial Design

The STEM trial is a single-center, two-phase, open-label, parallel randomized trial with three arms (2% soymilk, 2% cow’s milk, and SSBs). It assesses the effects of replacing SSBs (control) with either 2% soymilk (the “active intervention”) or 2% cow’s milk (the standard of care or “reference intervention”) on IHCL, measured by proton magnetic resonance spectroscopy (^1^H-MRS), and on a range of cardiometabolic risk factors over 24 weeks. Participants are adults who are habitual consumers of SSBs (≥1 SSB/day) and living with obesity (high body mass index [BMI] and high waist circumference). The trial is conducted in an outpatient setting at the Clinical Nutrition and Risk Factor Modification Centre at St. Michael’s Hospital (Toronto, ON, Canada). The trial protocol conforms to the ethical guidelines of the Tri-Council Policy Statement 2 [46], and the study is being conducted in accordance with the Declaration of Helsinki. Ethical approval was obtained by the St. Michael’s Hospital Research Ethics Board (protocol #21-172, 24 September 2021), and all participants provided written informed consent. The trial is registered on ClinicalTrials.gov (NCT05191160, 2 November 2021), and the full protocol is available in Supplementary File S1.

#### 2.1.1. Inclusion and Exclusion Criteria

Table 1 shows the finalized inclusion and exclusion criteria. A series of protocol amendments was made between 30 November 2021 and 16 August 2023 to improve recruitment feasibility (summarized in Supplementary Table S2). Participants were eligible if they reported consuming at least one 355 mL serving of a sugar-sweetened beverage (SSB) per day. Additional inclusion criteria included age between 18 and 75 years, a BMI classified as overweight or obese (BMI ≥ 25 kg/m^2^), and an elevated waist circumference based on ethnic-specific cut-offs [47]. Key exclusion criteria included a self-reported allergy or intolerance to soy or cow’s milk; any condition or circumstance that would prevent safe participation in a ^1^H-MRS scan; uncontrolled hypertension or severe hypertriglyceridemia; and other self-reported exclusionary conditions or diagnoses.

#### 2.1.2. Randomization and Allocation Concealment

Randomization was performed following successful completion of the run-in phase. Block randomization was conducted by an off-site statistician at the Applied Health Research Centre (AHRC) at St. Michael’s Hospital using the Research Data Capture (REDCap) platform with allocation concealment. Participants were randomized to three groups from within each stratum using computer-generated permuted blocks of unequal size. Allocation concealment was maintained through secure electronic delivery of a single assignment sequence for each consecutively enrolled participant.

#### 2.1.3. Interventions

Table 2 lists the three study beverages: 2% soymilk (250 mL, 100 kcal, 8 g soy protein, and 6 g sugars per serving), 2% cow’s milk (250 mL, 130 kcal, 8 g casein/whey protein, and 12 g sugars per serving), and SSBs (355 mL, 130–140 kcal, 0 g protein, and 32–41 g of sugars per serving). To allow for pragmatic beverage replacement using commercially available products, the study beverages were not isocaloric. However, the “active intervention” (2% soymilk) and “reference intervention” (2% cow’s milk) were matched for serving size and protein content. Although the soymilk and cow’s milk beverages were not matched for sugar content, the soymilk is an iso-sweet analogue of cow’s milk, as sucrose is approximately 1.4 times sweeter than lactose [49].

The prescribed daily dose of the study beverages for each participant is matched to their baseline SSB intake (in 355 mL servings), allowing for a direct replacement and a natural dose response. The maximum allowable intake is six servings per day to prevent excessive intake and ensure that total soy isoflavone exposure does not exceed the upper amount typically used in intervention studies or the upper intake observed in free-living populations [50,51]. Participants randomized to the 2% soymilk or 2% cow’s milk arms are instructed to replace their usual SSBs with the assigned study beverage, while participants in the control group are instructed to continue drinking their usual SSBs [50,51]. All study beverages are provided by the research team.

#### 2.1.4. Blinding

Blinding of the participants and trial personnel was not feasible due to the nature of the intervention. However, outcome assessors and statisticians will remain blinded to the treatment allocation.

#### 2.1.5. Recruitment

Participants were recruited from the Greater Toronto Area through multiple channels: outreach by study staff through an internal list of previous trial participants, internally-run social media advertisements (Facebook and Instagram), online classifieds (Craigslist and Kijiji), community postings at St. Michael’s Hospital, the University of Toronto, local healthcare clinics, and public venues (e.g., grocery stores, recreation centers, convenience stores, malls, drug stores, places of worship, and community bulletin boards), Canada Post direct mailings, and advertising by external digital marketing organizations (Trialfacts and Leapcure [formerly Honeybee Trials]).

#### 2.1.6. Trial Protocol

**Figure 1:**
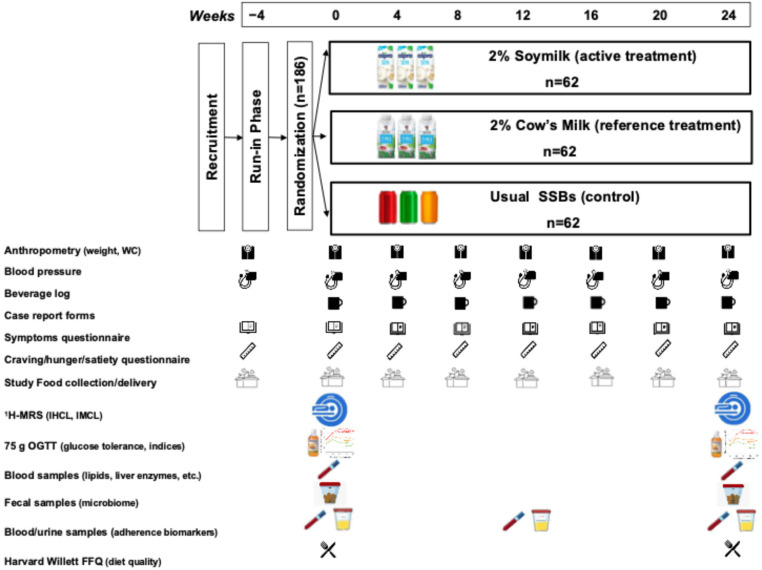
Trial Protocol. SSB = sugar-sweetened beverage; WC = waist circumference; ^1^H-MRS = proton magnetic resonance spectroscopy; IHCL = intrahepatocellular lipid; IMCL = intramyocellular lipid; 75 g OGTT = 75 g oral glucose tolerance test; Harvard Willet FFQ = Harvard Willet Food Frequency Questionnaire.

Figure 1 shows the protocol and data collection schedule. Participants will attend the Clinical Nutrition Risk Factor Modification Centre and Canadian Foundation of Innovation (CFI)-funded MR Research Unit at St. Michael’s Hospital on an outpatient basis. After providing informed consent and completing in-person screening, eligible participants were enrolled in a four-week run-in phase, during which they continued to consume their usual SSBs. Following the run-in, participants were randomized to one of three study arms and instructed to maintain their background diet and physical activity routines throughout the 24-week intervention. The intervention phase includes seven study visits. Prior to the week 0 and week 24 visits, participants are instructed to consume at least 150 g of carbohydrate for three consecutive days, fast for 10–12 h overnight, and collect both a stool sample (Stool Nucleic Acid Collection and Preservation System [Norgen Biotek Corp., Thorold, ON, Canada]) and a 24-h urine sample. At these visits, participants undergo a 30-min ^1^H-MRS scan and a 2-h 75 g oral-glucose tolerance test (75 g OGTT), following standard protocols [52]. Across all study visits, participants complete questionnaires and anthropometric assessments. Breakfast is provided following the procedures, and participants receive compensation for travel and time. To support adherence between visits, participants are provided with beverage logs for tracking intake, receive home delivery of study beverages, and participate in bi-weekly motivational phone calls. Participants with anxiety or claustrophobia related to the ^1^H-MRS scan are offered an oral anxiolytic.

### 2.2. Outcomes

Supplementary File S1 includes a detailed description of the methods for assessing the primary, secondary, exploratory, adherence, and safety outcomes.

#### 2.2.1. Primary Outcome

The primary outcome is the change in IHCL at week 24. IHCL will be measured by ^1^H-MRS using standard techniques [53] at the Canadian Foundation of Innovation (CFI)-funded MR Research Unit at St. Michael’s Hospital using a 3T Siemens Magnetom Skyra scanner (Siemens Healthineers, Erlangen, Germany).

#### 2.2.2. Secondary Outcomes

Secondary outcomes are key markers of glucose and insulin regulation derived from a 2-h 75 g OGTT at week 24. These markers include whole-body insulin sensitivity (Matsuda insulin sensitivity index [ISI] [54]), beta-cell function (insulin secretion-sensitivity index-2 [ISSI-2] [55,56]), and glucose tolerance (plasma glucose area under the curve [AUC] and 2-h plasma glucose [2 h-PG] [57,58,59]). Analyses will be performed using standard techniques [60,61,62] at Mount Sinai Services, Toronto, on the Roche Cobas c 502 system (Roche Diagnostics, Basel, Switzerland).

#### 2.2.3. Exploratory Outcomes

Exploratory outcomes are anthropometric, cardiometabolic, microbiomic, and dietary markers assessed at week 24. These outcomes include body weight, ectopic muscle fat (intramyocellular lipid [IMCL] in calf muscles by ^1^H-MRS [53], metabolic syndrome (MetS) criteria (waist circumference [63], systolic blood pressure [SBP], diastolic blood pressure [DBP] [64], fasting plasma glucose [FPG] [61,62], triglycerides [TG] [65], high-density lipoprotein cholesterol [HDL-C] [64,66,67]), revision of MetS criteria [47], type 2 diabetes incidence (Diabetes Canada 2018 FPG and 2 h-PG or hemoglobin A1C [HbA1c] criteria [68]), microbiome diversity [69,70,71], metabolic dysfunction-associated steatotic liver disease (MASLD) markers (alanine aminotransferase [ALT] [72,73], aspartate aminotransferase [AST] [72,73], gamma-glutamyl transferase [GGT] [74], and alkaline phosphatase [ALP] [75], fatty liver index [FLI] [76]), reversion of MASLD [77,78,79,80], hepatic insulin resistance (Homeostatic Model Assessment of Insulin Resistance [HOMA-IR] [81,82]), uric acid [83], established lipid targets (low-density lipoprotein cholesterol [LDL-C] [84,85], non-HDL-C, total cholesterol [TC] [86,87]), inflammation (high sensitivity *C*-reactive protein [hs-CRP] [88,89]), kidney function (creatinine [90], estimated glomerular filtration rate [eGFR] [91], urinary albumin excretion rate [AER] [92], albumin-to-creatinine ratio [ACR]), diet quality (Alternative Healthy Eating Index [AHEI] via the Harvard Food Frequency Questionnaire [FFQ] [93]), and appetite, food craving control, food cravings (savory and sweet), perceived craving control, and positive mood by the Control of Eating Questionnaire (CoEQ) [94]. Anthropometric measurements are done at the Clinical Nutrition Risk Factor Modification Centre. ^1^H-MRS measurements will be done at the Canadian Foundation of Innovation (CFI)-funded MRI Research Unit at St. Michael’s Hospital using a 3T Siemens Magnetom Skyra scanner (Siemens Healthineers). Laboratory measurements will be done at Mount Sinai Services, Toronto, on the Roche Cobas c 502 system (Roche Diagnostics).

#### 2.2.4. Adherence Outcomes

Adherence outcomes are objective biomarkers of each study beverage and self-reported intake of the study beverages at week 24. Objective biomarkers will be measured in the Department of Nutritional Sciences at the University of Toronto by established methods: urinary isoflavones excretion (UIE) for 2% soymilk [95,96], dairy-derived serum fatty acids (15:0, 17:0, conjugated linoleic acid [CLA], trans-palmitoleic acid [TPA]) for 2% cow’s milk [97,98,99], and urinary sucrose and fructose for SSBs [100,101,102]. Self-reported beverage intake is measured using beverage logs.

#### 2.2.5. Safety Outcomes

Safety outcomes are total self-reported symptoms and adverse events over 24 weeks. Symptoms are measured using a symptoms questionnaire [103]. Adverse events are measured by case report forms.

### 2.3. Statistical Analysis

All statistical analyses will be assessed at week 24 using STATA 17 (StataCorp, College Station, TX, USA). The primary analysis will be by the intention-to-treat (ITT) principle using inverse probability weighting (IPW) [104] for superiority testing with non-inferiority testing by per-protocol analyses. ITT analyses will include all randomized participants, and per-protocol analyses will include only those considered to be adherent to the study protocol. Secondary analyses will be conducted by completers and per-protocol. Hierarchical testing will be conducted for the primary outcome with superiority testing of the “reference intervention” (2% cow’s milk) compared to the control (SSB) tested first, followed by the “active intervention” (2% soymilk) compared to the control (SSB). If the superiority of 2% cow’s milk and 2% soymilk over SSBs is demonstrated (establishing the assay sensitivity), then the non-inferiority of 2% soymilk compared to 2% cow’s milk will be tested using per-protocol analysis. Non-inferiority will be established if the upper bound of the 90% confidence interval (CI) of the difference of means is less than the 1.5% non-inferiority margin. The parallel gatekeeping procedure will be used to control the family-wise error rate between the primary and secondary outcomes. If the superiority of 2% cow’s milk and 2% soymilk over SSBs is demonstrated (establishing the assay sensitivity), then the secondary outcomes will be analyzed using the Benjamini-Hochberg false discovery rate controlling procedure [105]. This method transfers any unused portion of the α from the primary outcome to the secondary outcomes if the primary outcome is statistically significant. If the primary outcome is not statistically significant in superiority testing, then secondary outcomes will be treated as hypothesis-generating without adjustment for false discovery. All other outcomes (exploratory, adherence, safety outcomes) will be tested without adjustment for false discovery, irrespective of the results of the testing of the primary outcome. Continuous data will be analyzed using analysis of covariance (ANCOVA), and categorical data will be analyzed using logistic regression and the chi-square test at a significance level of *p* < 0.05. All models will be adjusted for age, sex, MASLD status, medication use, intervention dose (servings/day), and baseline level [106]. Continuous linear and non-linear dose response analyses will be conducted over the natural dose range (1–6 servings/day) using multiple linear regression and piecewise regression, respectively. Prespecified subgroup analyses will be conducted using Chi-square tests by age, sex, MASLD status, caffeinated beverage use, intervention dose (servings/day), baseline MetS criteria (waist circumference, FPG, TG, HDL-C, systolic blood pressure, and diastolic blood pressure), and baseline values of the outcome being assessed.

### 2.4. Power Calculation

Table 3 presents the power calculations for non-inferiority and superiority testing of the primary outcome, as well as for superiority testing of the secondary outcomes. It was determined that 186 participants (N = 62 per arm) would be required to test the non-inferiority of the active intervention (2% soymilk) compared with the reference intervention (2% cow’s milk) based on a non-inferiority margin [δ] of 1.5% absolute difference in IHCL, and to test the superiority of the active and reference interventions compared with the control (SSBs), assuming a 2% absolute difference in IHCL. The selected cut-offs were chosen based on FDA and European Medicines Agency guidance [107,108]. A 2% absolute difference in IHCL is the minimum absolute reduction seen in nutrition and lifestyle interventions that is associated with clinically meaningful changes in downstream glycemic control, insulin sensitivity, blood pressure, and/or blood lipids [109,110,111,112,113,114,115]. A non-inferiority margin of 1.5% in IHCL was selected based on the minimum relative difference associated with benefit (30%) at the minimum absolute MASLD cut-off of 5% [116], which equates to a 1.5% absolute difference [117,118,119,120,121]. The sample size provides ≥80% power for both primary outcome comparisons, assuming a standard deviation (SD) of 3%, alpha (α) of 0.05, and 20% attrition. The sample size will also provide adequate power for superiority testing of the secondary outcomes (Matsuda ISI, ISSI-2, plasma glucose AUC, and 2 h-PG), using parallel gatekeeping and the Benjamini-Hochberg procedure with parallel gatekeeping for control of false discovery [105].

## 3. Results

### 3.1. CONSORT Statement

**Figure 2:**
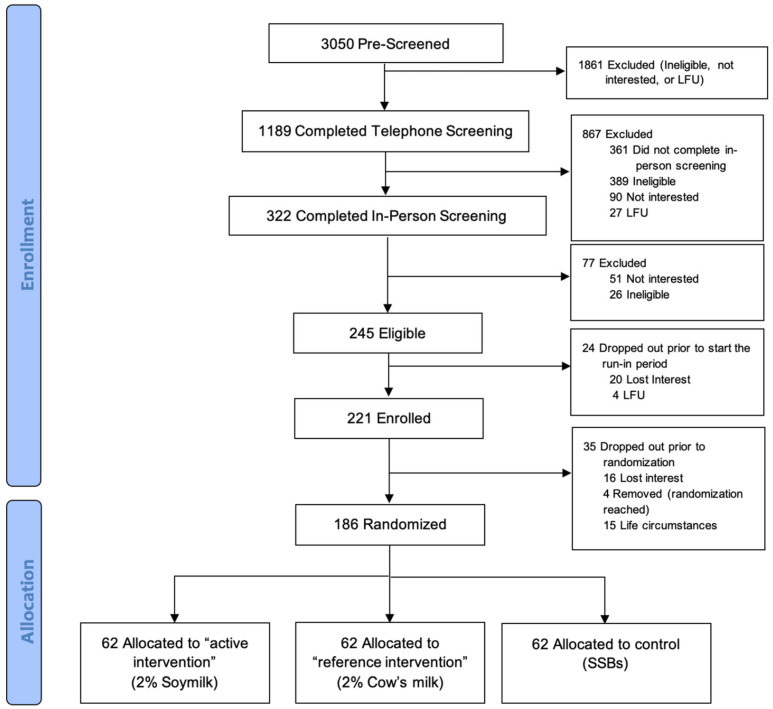
CONSORT Statement, LFU = lost to follow-up.

Figure 2 shows the CONSORT diagram for the trial. Recruitment began in November 2021, and all participants were randomized between 19 April 2022 and 16 April 2024. A total of 3050 individuals were pre-screened, 1189 completed the telephone screening, and 322 provided written informed consent and completed the in-person screening. Of these, 245 individuals were eligible to participate, and 221 enrolled. The reasons for not enrolling were loss of interest (N = 20) and loss to follow-up (N = 4). Following enrollment, 35 participants withdrew prior to randomization due to loss of interest (N = 16), life circumstances (N = 15), and were removed from the trial after completing the randomization phase (N = 4). Therefore, a total of 186 participants were randomized (N = 62 per arm).

### 3.2. Baseline Characteristics

Table 4 presents the baseline characteristics of participants randomized to the “active intervention” (2% soymilk; N = 62), “reference intervention” (2% cow’s milk; N = 62), and control (SSB; N = 62) arms. There were no statistically significant differences in baseline characteristics across the three groups.

Participants were predominantly male (57%) with a mean age of 39.9 years (mean age, 39.9 years ± 11.8 years), a mean BMI in the obese range (mean BMI, 34.6 kg/m^2^ ± 6.1 kg/m^2^), and an elevated waist circumference (mean waist circumference, 112.6 cm ± 13.8 cm). Mean systolic and diastolic blood pressures were within normal ranges (mean SBP, 118.34 mmHg ± 15.6 mmHg and mean DBP, 75.83 mmHg ± 11.4 mmHg). At baseline, mean IHCL was 10.0% ± 8.2%, with 64.1% of participants (N = 184) meeting the criteria for MASLD (IHCL ≥ 5% plus 1 risk factor [77,78,79,80]). Two participants dropped out of the study prior to the baseline scan.

Participants were primarily Caucasian (38.7%), with most having completed high school (29.57%), college (30.1%), or an undergraduate degree (26.9%). Nearly half (45.2%) were employed full time. Among participants who reported consuming alcohol, most did so on either a monthly (19.9%) or weekly (12.4%) basis.

Relevant self-reported medication use included antihyperlipidemics (5.9%), antihypertensives (9.1%), antihyperglycemics (2.7%), antidepressants (21%), antipsychotics (7.5%), anti-inflammatories (3.2%), anticonvulsants (2.2%), antiretrovirals (1.6%), psychostimulants (4.3%), oral contraceptives (1.6%), thyroid hormone-replacement therapy (6.5%), gender-affirming hormone therapy (2.2%), and allopurinol (1%). Relevant supplement use included psyllium fiber (2.2%) and fish oil (3.3%).

Participants reported baseline consumption of 2.3 servings (355 mL) of SSBs per day, most commonly Coca-Cola (43%) and ginger ale (Canada Dry or Schweppes) (31.2%).

## 4. Discussion

### 4.1. Summary

The STEM trial is a pragmatic, three-arm, parallel, randomized trial evaluating both the superiority of 2% soymilk and 2% cow’s milk over SSBs, and the non-inferiority of 2% soymilk to 2% cow’s milk as a replacement strategy for SSBs on liver fat and key cardiometabolic mediators/markers over 24 weeks in adults who are habitual consumers of SSBs (≥1 SSBs/day) and living with obesity (high BMI and high waist circumference). Baseline characteristics were balanced across the three study arms. The cohort represents a population at elevated cardiometabolic risk, as evidenced by a mean BMI in the obese range, high waist circumference, and 64.1% meeting the criteria for MASLD at baseline. Population estimates in North America indicate that approximately 30–40% [122,123] have obesity and 25% meet the criteria for MASLD [124,125]. Although baseline mean systolic and diastolic blood pressure values were within the normal range, these values include participants taking antihypertensive agents. The most frequently reported relevant medications include antidepressants and antihyperlipidemic agents, while relevant supplement use (e.g., psyllium fiber and fish oil) was low. At baseline, mean SSB intake was 2.3 per day (355 mL each), with Coca-Cola being the most consumed beverage.

### 4.2. Strengths and Limitations

The present trial has several notable strengths. The 6-month intervention period will allow for a robust assessment of the longer-term effects on cardiometabolic risk factors. Regular intermediate study visits and scheduled telephone calls will support participant retention and adherence throughout the intervention. The sample size provides adequate statistical power for all planned comparisons, and the stepwise approach to non-inferiority and superiority testing will minimize the familywise error rate. The use of ITT analysis is considered the gold standard for clinical trials [126].

The use of commercially available, single-serve study beverages will enhance the generalizability and real-world applicability of the findings. By restricting enrollment to participants with markers associated with excess liver fat, such as overweight or obesity and elevated waist circumference, the study population reflects a group at elevated risk for type 2 diabetes and other cardiometabolic diseases. This at-risk phenotype enhances the external validity of the trial, as these individuals represent a substantial proportion of the North American population. The results will be relevant to policymakers, the food industry, and consumers of SSBs, cow’s milk, and plant-based dairy alternatives by providing clarity amid competing recommendations. The use of objective biomarkers to validate self-reported beverage intake reduces reporting bias and strengthens adherence assessment. Third-party randomization and data management further minimize the risk of bias.

Several limitations must be acknowledged. The COVID-19 pandemic introduced challenges both internal (e.g., hospital policies related to staff, personal protective equipment shortages, interruptions to research operations) and external (e.g., city-wide lockdowns and public hesitancy around visiting healthcare facilities) challenges [127,128]. These factors affect recruitment, daily operations, and protocol adherence. Nevertheless, study staff remain flexible in adapting to evolving policies while prioritizing protocol fidelity. The lengthy intervention phase also poses adherence challenges; however, this will be accounted for through per-protocol analysis informed by objective biomarkers of beverage intake.

Due to the nature of open-label randomized trials, participants may consciously or unconsciously modify other lifestyle behaviors in response to the interventions, despite instructions to maintain their usual diet and physical activity [129]. Such behavioral compensation may influence study outcomes. Any unintended lifestyle changes will be captured through study measures and will be explored in subgroup and sensitivity analyses to contextualize potential effects on study outcomes.

### 4.3. Implications

The findings of the STEM trial will have important implications for public health policy and dietary guidance. Demonstrating the role of fortified soymilk as a healthy, plant-based alternative to cow’s milk in strategies to reduce SSB consumption is highly relevant for both public and planetary health, particularly in the prevention and management of cardiometabolic disease. As a rigorously conducted, 24-week SSB-reduction randomized controlled trial, this study offers direct evidence to inform dietary guidelines and the FDA’s proposed “healthy” food criteria. Moreover, these findings may help clarify the applicability of the NOVA classification system to nutrient-rich soy-based foods.

## 5. Conclusions

The STEM trial is a pragmatic investigation to assess the longer-term effects of replacing SSBs with 2% soymilk or 2% cow’s milk on liver fat and other cardiometabolic risk factors in adult SSB consumers with obesity. A total of 186 participants (62 per arm) meeting the inclusion criteria were successfully recruited and randomized to the “active intervention” (2% soymilk), “reference intervention” (2% cow’s milk), and control (SSB) arms. Baseline characteristics were balanced across groups and confirm that the cohort represents adults with a high-risk metabolic phenotype, with the majority meeting the criteria for MASLD. This head-to-head comparison of soymilk and cow’s milk as replacements for SSBs will provide insights into their roles in SSB-reduction strategies and aid in clarifying soymilk’s role as a healthy plant-based alternative to cow’s milk for adults with obesity. Results will be disseminated through scientific presentations, peer-reviewed publications, and integration into systematic reviews and meta-analyses. Ultimately, the findings will inform clinical practice, support innovation in the food industry, and shape public health policies to reduce SSB consumption and promote plant-based dietary patterns.

## Figures and Tables

**Table 1:** Inclusion and Exclusion Criteria.

Inclusion Criteria	Exclusion Criteria
Adults (age 18–75 years), men, and non-pregnant women Overweight or obese (body mass index [BMI] ≥ 25 kg/m^2^) ^a^ High waist circumference (United States of America [USA]/Canada ≥102 cm in men, ≥88 cm in women; Europid/Caucasian/Middle East, Mediterranean/Sub-Saharan African ≥ 94 cm in men, ≥80 cm in women; and Asian [including Japanese]/Ethnic Central and South American ≥ 90 cm in men, ≥80 cm in women, with a waist diameter ≤60 cm) ^a^ [47,48] Regularly drinking sugar-sweetened beverages [SSB] (≥one 355 mL serving/day) ^b^	Uncontrolled hypertension (systolic blood pressure [SBP] ≥ 180 mmHg or diastolic blood pressure [DBP] ≥ 110 mmHg ^a^ Diabetes Cow’s milk or soy intolerance or allergy Pregnant or breastfeeding individuals, or individuals planning on becoming pregnant, throughout the study period Weight loss of ≥10% in the last 6 months Complementary or alternative medicines used are deemed inappropriate by investigators Wilson’s disease Haemochromatosis Inborn errors of metabolism Lipodystrophy Cushing syndrome or disease Gastrointestinal disease (inflammatory bowel disease or malabsorption disorder) Previous bariatric surgery Alcoholic fatty liver disease, cirrhosis, hepatocellular carcinoma, Hepatitis C, Hepatitis B, or Hepatitis A viral infections, or genetic causes of liver disease (Alpha-1-antitrypsin deficiency) Uncontrolled hyperthyroidism or hypothyroidism High risk or very high-risk chronic kidney (KIDIGO 2012 criteria) Acute or chronic infection (e.g., salmonellosis, human immunodeficiency virus, tuberculosis) Chronic inflammatory conditions Chronic lung disease Chronic pancreatitis or pancreatic insufficiency Cystic fibrosis Cancer/malignancy in the last 6 months, except skin cancer Schizophrenia spectrum and other psychotic disorders, bipolar and related disorders, and dissociative disorders Severe depression Major surgery in the last 6 months Hypopituitarism Self-reported hypogonadism Substance abuse disorder (including alcohol or recreational drugs) Participation in any intervention trials within the last 3 months or for the duration of this study Any condition or circumstance that would prevent having a proton magnetic resonance spectroscopy (^1^H-MRS) scan (e.g., pacemaker, neurostimulators, breast tissue expanders, implants, or foreign metal objects in the body) Individuals planning on making dietary or physical activity changes throughout the study duration If self-reported medication use, it must be at a stable dose for ≥6 months

BMI = body mass index; USA = United States of America; SSB = sugar-sweetened beverage; SBP = systolic blood pressure; DBP = diastolic blood pressure; KDIGO = Kidney Disease: Improving Global Outcomes; ^1^H-MRS = proton magnetic resonance spectroscopy. All criteria were self-reported unless otherwise indicated. Certain health conditions were deemed to be non-exclusionary when managed long-term (≥6 months) with medication or in remission, as determined by the primary investigator. ^a^ Not self-reported. ^b^ For screening purposes only, SSBs were defined to include soft drinks, sports/energy drinks, sweetened iced tea, and homemade beverages such as frescas or fruit drinks. This definition did not include sweetened coffee beverages or 100% fruit juice.

**Table 2:** List of Intervention Beverages.

Study Arm	2% Soymilk Group (“Active Intervention”)	2% Cow’s Milk Group (“Reference Intervention”)	SSB Group (Control)
**Brand**	Alpro^®^ Soya	Organic Meadow	Any soft drink available in the Canadian market ^a^
**Serving Size (mL)**	250 mL	250 mL	355 mL
**Energy (kcal) per Serving**	100 kcal	130 kcal	130–140 kcal
**Sugars per Serving**	6 g	12 g	32–41 g ^b^
**Protein per Serving**	8 g	8 g	0 g
**Packaging/** **Processing** **Specifications**	Single-serve, shelf-stable	Single-serve, shelf-stable	Single-serve can
**Ingredients**	Soya base (water, hulled soya beans [8%]), sugar, acidity regulators (potassium phosphates), calcium carbonate, flavoring, sea salt, stabilizer (gellan gum), vitamins (B2, B12, D2)	Organic partly skimmed milk, vitamin A palmitate, vitamin D_3_	Coca-Cola: Carbonated water, sugar/glucose-fructose, caramel color, phosphoric acid, natural flavor, caffeine Canada Dry Ginger Ale: Carbonated water, sugar/glucose-fructose, citric acid, sodium benzoate, color, natural flavor ^c^

SSB = sugar-sweetened beverage; mL = milliliter; kcal = kilocalorie; g = grams. ^a^ Must contain ≥50 kcal per 355 mL serving. ^b^ Range in quantity of sugars (g) across all SSB types used in the study. ^c^ Ingredient lists for the two most commonly consumed SSBs at baseline.

**Table 3:** Power (Sample Size) Calculation.

Outcome Type	Outcome	Mean Change	SD	Alpha A	N	Power (1-Beta) for Alpha 1 ^a^	Alpha 2 ^b^	Power (1-Beta) for Alpha 2
Primary (non-inferiority)	IHCL	1.5%	3%	0.05	62 in each arm	80%	-	
Primary (superiority)	IHCL	2%	3%	0.05	62 in each arm	91%	-	
Secondary (superiority)	Matsuda ISI	10%	15%	0.05	62 in each arm	91%	0.0125	80%
Secondary (superiority)	ISSI-2	10%	15%	0.05	62 in each arm	91%	0.0125	80%
Secondary (superiority)	Glucose iAUC	10%	15%	0.05	62 in each arm	91%	0.0125	80%
Secondary (superiority)	2 h-PG	10%	15%	0.05	62 in each arm	91%	0.0125	80%

IHCL = intrahepatocellular lipid, ISI = insulin sensitivity index, ISSI-2 = insulin secretion-sensitivity index-2, AUC = area under the curve, 2 h-PG = 2-h plasma glucose. ^a^ Power was corrected for 20% attrition. ^b^ Alpha 2 = worst case scenario is based upon Benjamini-Hochberg false discovery control, which in this case leads to a worst alpha of 0.0125 for the 4th *p*-value if all previous *p*-values have failed [alpha/m or 0.05/4 = 0.0125].

**Table 4:** Baseline Characteristics.

Variable	“Active Intervention” Arm (N = 62) (2% Soymilk)	“Reference Intervention” Arm (N = 62) (2% Cow’s Milk)	Control Arm (N = 62) (SSB)	*p*-Value
**Anthropometric Measures, Mean ± SD**				
Age (years)	41.31 ± 11.88	41.49 ± 12.07	43.11 ± 13.34	0.29
Females, N (%)	27 (44)	31 (50)	22 (36)	0.26
Height (cm) ^a^	169.42 ± 11.20	167.67 ± 10.01	169.73 ± 8.98	0.10
Weight (kg)	102.86 ± 24.54	97.54 ± 20.30	98.90 ± 19.33	0.36
BMI (kg/m^2^)	35.48 ± 5.96	34.05 ± 5.63	34.17 ± 6.62	0.35
Waist Circumference (cm)	114.19 ± 15.65	111.01 ± 12.90	112.55 ± 12.62	0.44
SBP (mmHg) ^b^	117.80 ± 15.69	118.46 ± 14.46	118.74 ± 16.92	0.94
DBP (mmHg) ^b^	75.66 ± 10.75	75.78 ± 10.55	76.10 ± 13.00	0.98
**Liver Fat ^c,d,e^**				
MASLD (IHCL ≥ 5% ± 1 risk factor) ^f^ N (%)	34 (56)	43 (69)	41 (67)	0.24
Mean IHCL %, Mean ± SD	9.54 ± 9.04	10.07 ± 7.26	10.52 ± 8.42	0.23
**Self-Reported Demographics, N (%)**				
**Ethnicity**				0.94
Caucasian	23 (37)	26 (42)	23 (37)	
Black	10 (16)	7 (11)	8 (13)	
Indigenous	2 (3)	1 (2)	2 (3)	
South Asian	10 (16)	8 (13)	12 (19)	
Middle Eastern/Arab	1 (2)	2 (3)	0 (0)	
West Asian	0 (0)	0 (0)	1 (2)	
Filipino	1 (2)	3 (5)	3 (5)	
South East Asian	0 (0)	2 (3)	1 (2)	
Latin American	4 (7)	3 (5)	4 (7)	
East Asian	6 (10)	3 (5)	3 (5)	
Mixed Ethnicities	5 (8)	7 (11)	5 (8)	
**Education**				0.63
Master’s degree	7 (11)	9 (15)	8 (13)	
Undergraduate degree	20 (32)	13 (21)	17 (27)	
College certificate or diploma	14 (23)	22 (36)	20 (32)	
High school or high school equivalent	21 (34)	17 (27)	17 (27)	
Did not complete high school	0 (0)	1 (2)	0 (0)	
**Work Status**				0.29
Full-time employee	31 (50)	29 (47)	24 (39)	
Part-time employee	9 (15)	10 (16)	14 (23)	
Casual employee	2 (3)	3 (5)	0 (0)	
Disability	8 (13)	4 (7)	6 (10)	
Retired	1 (2)	0 (0)	3 (5)	
Stay-at-home parent	2 (3)	5 (8)	3 (5)	
Unemployed	5 (8)	5 (8)	7 (11)	
Full-time student	0 (0)	1 (2)	3 (5)	
Part-time student	4 (7)	2 (3)	1 (2)	
Multiple work status	0 (0)	3 (5)	1 (2)	
**Alcohol Intake ^g^**				0.15
1 to 2 times per week	6 (10)	17 (27)	12 (19)	
1 to 2 times per month	16 (26)	9 (15)	12 (19)	
Every 2 to 3 months	9 (15)	8 (13)	6 (10)	
1 to 2 times per year	2 (3)	6 (10)	8 (13)	
Do not know	0 (0)	1 (2)	0 (0)	
**Self-Reported Medication Users, N (%) ^g^**				
Antihyperlipidemic (Statins)	5 (8)	1 (2)	5 (8)	0.21
Antihypertensive (ACEi N = 11, Diuretic N = 7, ARB N = 6, Ca+ Channel Blocker N = 12, Beta Blocker N = 4, Alpha Blocker N = 2, ARNI N = 1)	7 (11)	11 (18)	9 (15)	0.59
Antihyperglycemic (Biguanide N = 4, GLP-1 Agonist N = 1)	2 (3)	2 (3)	1 (2)	0.81
Antidepressant (SSRI N = 22, SNRI N = 10, NDRI N = 7, TCA N = 3, SARI N = 2, MAOi N = 1)	13 (21)	14 (23)	12 (19)	0.91
Antipsychotic	7 (11)	4 (7)	3 (5)	0.37
Anti-inflammatory (NSAID N = 6, Analgesic/Antipyretic N = 1)	1 (2)	2 (3)	3 (5)	0.60
Anticonvulsant	2 (3)	1 (2)	1 (2)	0.77
Antiretroviral (PReP N = 3)	0 (0)	1 (2)	2 (3)	0.36
Psychostimulant (Methylphenidate N = 3, Amphetamine N = 5)	5 (8)	2 (3)	1 (2)	0.18
Oral contraceptive	2 (3)	1 (2)	0 (0)	0.36
Thyroid HRT (T4 N = 12)	3 (5)	2 (3)	7 (11)	0.15
Gender-affirming hormone therapy (Estrogen N = 2, Testosterone N = 2)	1 (2)	2 (3)	1 (2)	0.77
Allopurinol	0 (0)	0 (0)	1 (2)	0.37
**Self-Reported Supplement Users, N (%)**				
Psyllium Fiber	1 (2)	3 (5)	0 (0)	0.17
Fish Oil	2 (3)	2 (3)	2 (3)	1.00
**SSB Intake, N (%) ^h^**				
**SSBs/day**				0.75
1	21 (34)	18 (29)	21 (34)	
2	21 (34)	23 (37)	21 (34)	
3	8 (13)	12 (19)	14 (23)	
4	6 (10)	5 (8)	1 (2)	
5	2 (3)	2 (3)	1 (2)	
6	4 (7)	2 (3)	4 (7)	
**Type of SSB**				0.21
*Caffeinated*				
Coca-Cola	27 (44)	26 (42)	27 (44)	
Pepsi	2 (3)	8 (13)	8 (13)	
Dr. Pepper	5 (8)	0 (0)	3 (5)	
*Non-Caffeinated*				
Ginger Ale	19 (30)	21 (34)	18 (29)	
Orange Crush	0 (0)	2 (3)	3 (5)	
Sprite	5 (8)	5 (8)	2 (3)	
7-Up	1 (2)	0 (0)	1 (2)	
Fanta Orange	1 (2)	0 (0)	0 (0)	
Root Beer	2 (3)	0 (0)	0 (0)	

SD = standard deviation; SSB = sugar-sweetened beverage; BMI = body mass index; SBP = systolic blood pressure; DBP = diastolic blood pressure; MASLD = metabolic dysfunction-associated steatotic liver disease; IHCL = intrahepatocellular lipid; ACEi = Angiotensin-Converting Enzyme inhibitor; ARB = Angiotensin II Receptor Blocker; ARNI = Angiotensin Receptor-Neprilysin Inhibitor; GLP-1 = Glucagon-Like Peptide-1 receptor agonist; SSRI = Selective Serotonin Reuptake Inhibitor; SNRI = Serotonin-Norepinephrine Reuptake Inhibitor; NDRI = Norepinephrine-Dopamine Reuptake Inhibitor; TCA = Tricyclic Antidepressant; SARI = Serotonin Antagonist and Reuptake Inhibitor; MAOi = Monoamine Oxidase Inhibitor; PrEP = Pre-Exposure Prophylaxis. ^a^ Height was measured at screening. ^b^ Mean systolic and diastolic blood pressure values include measurements from participants taking antihypertensive agents. ^c^ Measured at week 0 (baseline) study visit, calculated from the mean fat % of anterior, posterior, and mid lobes. ^d^ Two randomized participants dropped out of the study prior to completing the week 0 MRI. ^e^ MASLD diagnostic criteria [77,78,79,80]. ^f^ Answered only by participants who self-reported alcohol intake. ^g^ Some participants were taking multiple medications from the same category; ^h^ SSB consumption is based on self-reported average daily consumption at screening.

## Data Availability

The data presented in this study are available on request from the corresponding author due to ethical reasons.

## Supplementary Materials

The following supporting information can be downloaded at: https://www.mdpi.com/article/10.3390/nu18071026/s1, File S1: STEM Trial Protocol, File S2: Supplementary Tables.

## Abbreviations

SSB = sugar-sweetened beverage, UPF = ultra-processed food, STEM = Soy Treatment Evaluation for Metabolic Health, BMI = body mass index, IHCL = intrahepatocellular lipid, ^1^H-MRS = proton magnetic resonance spectroscopy, CI = confidence interval, ITT = intention-to-treat, ANCOVA = analysis of covariance, IPW = inverse probability weighting, SD = standard deviation, MASLD = metabolic dysfunction-associated steatotic liver disease, DGA = Dietary Guidelines for Americans, FDA = Food and Drug Administration, WHO = World Health Organization, NOVA = NOVA food classification system, AHRC = Applied Health Research Centre, REDCap = Research Electronic Data Capture, CFI = Canadian Foundation for Innovation, 75 g OGTT = 75 g oral glucose tolerance test, ISI = insulin sensitivity index, ISSI-2 = insulin secretion–sensitivity index-2, AUC = area under the curve, 2h-PG = 2-h plasma glucose, IMCL = intramyocellular lipid, MetS = metabolic syndrome, FPG = fasting plasma glucose, TG = triglycerides, HDL-C = high-density lipoprotein cholesterol, HbA1c = hemoglobin A1c, ALT = alanine aminotransferase, AST = aspartate aminotransferase, GGT = gamma-glutamyl transferase, ALP = alkaline phosphatase, FLI = fatty liver index, HOMA-IR = Homeostatic Model Assessment of Insulin Resistance, LDL-C = low-density lipoprotein cholesterol, non-HDL-C = non-high-density lipoprotein cholesterol, TC = total cholesterol, hs-CRP = high-sensitivity *C*-reactive protein, eGFR = estimated glomerular filtration rate, AER = albumin excretion rate, ACR = albumin-to-creatinine ratio, AHEI = Alternative Healthy Eating Index, FFQ = Food Frequency Questionnaire, SBP = systolic blood pressure, DBP = diastolic blood pressure, UIE = urinary isoflavone excretion, CLA = conjugated linoleic acid, TPA = trans-palmitoleic acid, α = alpha, STATA 17 = Statistical Software (StataCorp LLC, TX, USA), ANOVA = analysis of variance, ACEi = angiotensin-converting enzyme inhibitor, ARB = angiotensin II receptor blocker, ARNI = angiotensin receptor–neprilysin inhibitor, GLP-1 = glucagon-like peptide-1 receptor agonist, SSRI = selective serotonin reuptake inhibitor, SNRI = serotonin–norepinephrine reuptake inhibitor, NDRI = norepinephrine–dopamine reuptake inhibitor, TCA = tricyclic antidepressant, SARI = serotonin antagonist and reuptake inhibitor, MAOi = monoamine oxidase inhibitor, PrEP = pre-exposure prophylaxis.
