# Supplementary material for: Rationale, Design, and Participant Baseline Characteristics of a Parallel Randomized Trial of the Effect of Replacing SSBs with Cow’s Milk Versus Soymilk on Intrahepatocellular Lipid and Other Cardiometabolic Risk Factors in Adults with Obesity Who Consume Sugar-Sweetened Beverages: The Soy Treatment Evaluation for Metabolic health (STEM) Trial
Source: Nutrients. 2026 Mar 24;18(7):1026. doi: 10.3390/nu18071026 (PMC13074930; doi:10.3390/nu18071026)
Supplement: Supplementary file 1 [file nutrients-18-01026-s001.zip › nutrients-4173533-File S2.pdf]

Supplemental Material: Rationale, Design, and Participant Baseline Characteristics of a Parallel Randomized Trial of the Effect of Replacing SSBs with Cow's Milk versus Soymilk on Intrahepatocellular Lipid and other Cardiometabolic Risk Factors in Obese Adult SSB Consumers: The Soy Treatment Evaluation for Metabolic health (STEM) Trial

Erlich et al.

Table of Contents

Supplementary Table S1: 13 Countries with Documented Regulatory Health Claims for Soy Protein and Heart Health 2

Supplementary Table S2: Protocol Amendments Affecting Inclusion and Exclusion Criteria 4

Supplementary Table S1: 13 Countries with Documented Regulatory Health Claims for Soy Protein and Heart Health

Country	Approval Year	Regulatory Institution [a]	Claim Wording [b]	Minimum/ Serving	Daily Intake	Claim Type
Brazil	2005	Agência Nacional de Vigilância Sanitária (ANVISA)[1]	"Soy protein contributes to the reduction of cholesterol, which may reduce the risk of heart disease"	6.25 g	25 g	Disease Risk
Canada	2015	Health Canada[2]	"Soy protein helps reduce blood cholesterol"	6 g	25 g	Cholesterol Proxy
Chile	2006	Ministerio de Salud (MINSAL)[3]	"25 g of soy protein per day, as part of a diet low in saturated fat and cholesterol, may reduce the risk of coronary heart disease"	6.25 g	25 g	Disease Risk
Colombia	2008	Instituto Nacional de Vigilancia de Medicamentos y Alimentos (INVIMA)[4, 5]	"25 g of soy protein per day, as part of a diet low in saturated fat and cholesterol, may reduce the risk of cardiovascular disease"	6.25 g	25 g	Disease Risk
Japan	1996	Ministry of Health, Labour and Welfare (MHLW)[6]	"This product contains isolated soya protein, which helps decrease serum cholesterol level"	~3–7 g	6–7.8 g	Cholesterol Proxy
Malaysia	2006	Ministry of Health Malaysia[7]	"Soy protein helps to reduce cholesterol"	5 g	25 g	Cholesterol Proxy
South Africa	2002	Department of Health[8]	"Diets which contain at least 25 g soy protein (4 servings) daily… may reduce risk of heart disease by lowering cholesterol levels"	6.25 g	25 g	Disease Risk

South Korea	2005	Ministry of Food and Drug Safety (MFDS)[9]	"Helps maintain healthy blood cholesterol level"	-	15 g	Cholesterol Proxy
Turkey	2006	Ministry of Food, Agriculture and Livestock[10]	"This food contains soy protein. Soy protein helps lower cholesterol levels; low cholesterol helps maintain heart and vascular health"	6.25 g	25 g	Cholesterol Proxy
USA	1999	Food and Drug Administration (FDA)[11]	"25 g soy protein/day… may reduce risk of heart disease"	6.25 g	25 g	Disease Risk

Daily intake for cholesterol-lowering/heart health effect is 25 g soy protein/day in all countries except Japan (6–7.8 g/day) and South Korea (15 g/day), based on local regulatory standards and clinical evidence.; Minimum per serving varies (6–6.25 g in most countries; 5 g in Malaysia).; Claim Type: "Disease Risk" = direct link to reduced heart disease risk; "Cholesterol Proxy" = cholesterol-lowering as a scientifically accepted proxy for heart health.; [a] Reasoning for both original codification document cited: Colombia: 2008 Decree = supplements only; 2022 Acta = all foods. [b] Translated from the respective language into English. All sources are primary regulatory documents.

Supplementary Table S2: Protocol Amendments Affecting Inclusion and Exclusion Criteria

**Date of Approval**	**Change to STEM Trail Inclusion/Exclusion Criteria**
November 30, 2021	• Added uncontrolled hypertension as an exclusion criterion
February 8, 2022	• Expanded definition of SSBs to include sports and energy drinks, sweetened iced tea, and homemade SSBs<br>• Dysglycemia included under MetS criteria (now requires ≥2 MetS components rather than being a separate requirement)<br>• Removed claustrophobia as an exclusion criterion
April 12, 2022	• Changed SSB consumption criterion from ≥3 to ≥2 serving/day
May 26, 2022	• Changed SSB consumption criterion from ≥3 to ≥1 serving/day (with ≥50% of participants still required to consume ≥3 servings/day)<br>• Allowed inclusion of participants on certain medications if stable for ≥6 months<br>• Removed exclusion for prior colectomy or small bowel resection
March 20, 2023	• Removed upper BMI limit<br>• Added maximum waist circumference of ≤60 cm (maximum diameter to fit in the MRI scan) to inclusion criteria<br>• Modified MetS criteria: allowed inclusion with BMI + WC + 1 additional MetS component (instead of 2)
April 13, 2023	• Removed tobacco smoking as an exclusion<br>• Replaced exclusions for recreational drug use and heavy alcohol use with "self-reported substance use disorder"
May 23, 2023	• Added ethnic-specific WC cutoffs
August 16, 2023	• Removed MetS from inclusion criteria<br>• Removed requirement for additional MetS components beyond BMI and WC, eliminating the need for screening bloodwork
September 28, 2023	Removed atherosclerotic cardiovascular disease, Coeliac's disease and "other major illness or health related disease from exclusion criteria.

Abbreviations: STEM Trial = Soy Treatment Evaluation for Metabolic Health Trial; SSB = Sugar-Sweetened Beverage; MetS = Metabolic Syndrome; BMI = Body Mass Index; WC = Waist Circumference

References:

1. Sanitária, A.N.d.V., *Resolução RDC nº 2, de 7 de janeiro de 2005* 2005, ANVISA.
2. *Summary of Health Canada's Assessment of a Health Claim about Soy Protein and Cholesterol Lowering*, in *Health Products and Food Branch*, H. Canada, Editor. 2015, Health Canada: Ottawa.
3. Salud, M.d., *Normas Técnicas sobre Directrices Nutricionales – Exenta N° 764* 2006, MINSAL.
4. INVIMA, *Decreto 3863 de 2008 – Registro Sanitario de Suplementos Dietarios*. 2008, INVIMA.
5. INVIMA, *Acta No. 16 de 2022 – Comité de Alimentos: Aprobación de Alegaciones de Sağlık*. 2022, INVIMA.
6. Ministry of Health, L.a.W., *Foods for Specified Health Uses (FOSHU) – Soy Protein Approval History* 1996
7. Malaysia, M.o.H., *Food Regulations 1985 – Twelfth Schedule*. 2006, Ministry of Health
8. *Regulations Relating to the Labelling and Advertising of Foodstuffs: Government Notice R.1055* D.o. Health, Editor. 2002, Government Gazette. p. 1–60
9. Safety, M.o.F.a.D., *Health Functional Food Code* 2005.
10. Ministry of Food, A.a.L., *Türk Gıda Kodeksi Etiketleme Tebliği (2006/3)* 2006, Resmî Gazete Issue: 26057
11. Administration, F.a.D., *Food Labeling: Health Claims; Soy Protein and Coronary Heart Disease*. 1999. p. 57700–57733
