# Supplementary material for: Rationale, Design, and Participant Baseline Characteristics of a Parallel Randomized Trial of the Effect of Replacing SSBs with Cow’s Milk Versus Soymilk on Intrahepatocellular Lipid and Other Cardiometabolic Risk Factors in Adults with Obesity Who Consume Sugar-Sweetened Beverages: The Soy Treatment Evaluation for Metabolic health (STEM) Trial
Source: Nutrients. 2026 Mar 24;18(7):1026. doi: 10.3390/nu18071026 (PMC13074930; doi:10.3390/nu18071026)
Supplement: Supplementary file 1 [file nutrients-18-01026-s001.zip › nutrients-4173533-File S1.pdf]

PROTOCOL

Role of soy in metabolic health: The Soy Treatment Evaluation for Metabolic health (STEM) trial

Running Title: STEM trial
Clinical Trials Protocol Registration #: NCT05191160
REB #: 21-172

Principal Investigator:
John L. Sievenpiper, MD, PhD, FRCPC
Clinical Nutrition and Risk Factor Modification Centre,
St. Michael's Hospital, #6138-61 Queen St East.,
Toronto, ON, M5C 2T2, CANADA

Co-Investigators:
Lawrence A. Leiter, MDCM, FRCPC
Division of Endocrinology and Metabolism
St. Michael's Hospital, #6121-61 Queen St East,
Toronto ON, M5C 2T2, CANADA
Cyril W.C. Kendall, PhD
Department of Nutritional Sciences,
Temerty Faculty of Medicine, University of Toronto,
Medical Sciences Building, 1 King's College circle,
Toronto, ON, M5S 1A8, CANADA
David J.A. Jenkins, OC, MD, PhD, DSC, FRCP, FRCPC, FRSC
Clinical Nutrition and Risk Factor Modification Centre,
St. Michael's Hospital, #6131-61 Queen St East.,
Toronto, ON, M5C 2T2, CANADA
Richard Bazinet, PhD
Department of Nutritional Sciences,
Temerty Faculty of Medicine, University of Toronto,
Medical Sciences Building, 1 King's College circle,
Toronto, ON, M5S 1A8, CANADA

Study Coordinators:
Sonia Blanco Mejia, MD, MSc
Laura Chiavaroli, PhD
Clinical Nutrition and Risk Factor Modification Centre,
St. Michael's Hospital, #6139-61 Queen St E., Toronto, ON, M5C 2T2, CANADA

Sponsor: United Soybean Board (the United States Department of Agriculture [USDA] Soy "Check-off" Program)

Version Number: 18 | 19 NOV 2024

1. PROTOCOL SUMMARY

**Title:**	**Role of soy in metabolic health: The Soy Treatment Evaluation for Metabolic Health (STEM) trial**
**Short Title:**	STEM Trial
**REB Number:**	21-172
**Study Description:**	This is a 24-week, 2-phase, 3-arm, non-inferiority, open-label, parallel group, randomized controlled trial.
**Objectives:**	To assess the effect of 2% soy milk versus 2% cow's milk (casein and whey vehicle matched for protein and volume) as a "public health intervention" to replace sugar sweetened beverages (SSBs) on liver fat and key cardiometabolic mediators/indicators in metabolic syndrome (MetS).
**Endpoints:**	Primary outcome: liver fat.<br>Secondary outcomes: muscle fat, insulin sensitivity, beta-cell function, glucose tolerance and established cardiometabolic risk factors.
**Study Population:**	Overweight/obese participants with MetS who are consuming ≥1 SSBs/day
**Study Center:**	Clinical Nutrition and Risk Factor Modification Centre,<br>St. Michael's Hospital,<br>61 Queen St E., 6th Floor,<br>Toronto, ON, M5C 2T2, CANADA
**Study Interventions:**	1) 2% Soy milk,<br>2) 2% Cow's milk or<br>3) SSBs
**Figure 1. Design of the Soy Treatment Evaluation on the progression of Metabolic health (STEM)**	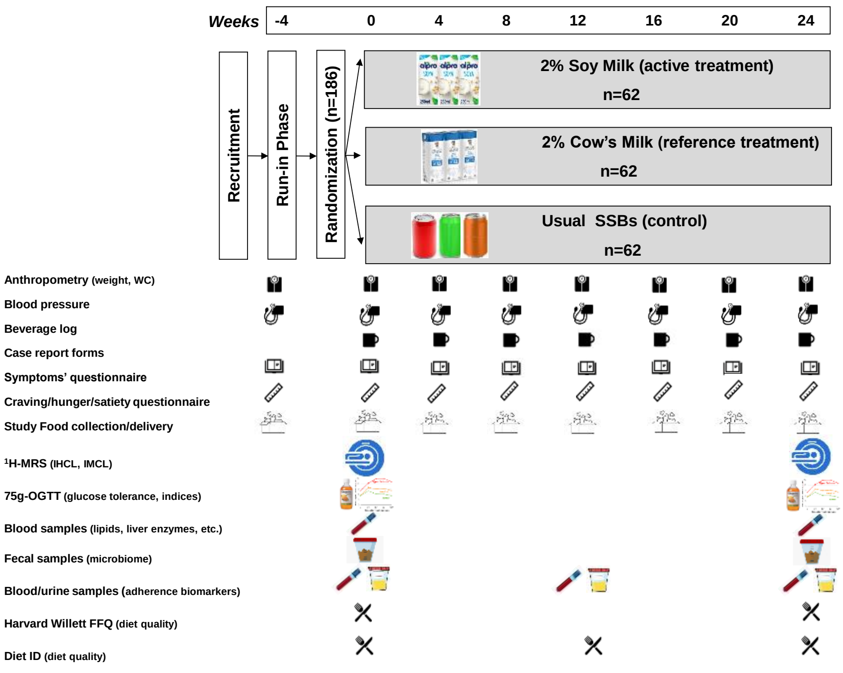
**Funding:**	United Soybean Board (USDA Soy "Check-off" Program)

2. BACKGROUND AND SIGNIFICANCE

Soy is at a nutritional "cross-roads". On one hand, it aligns with current dietary advice to consume plant-based dietary patterns [1-6] and has proven advantages [7-9]. On the other hand, it is under threat to have its health claim for coronary heart disease (CHD) risk reduction revoked [10] and is often overshadowed by dairy in public health interventions for metabolic health. Strategies to reduce sugar-sweetened beverages (SSB) have become one of the leading public health targets to address the epidemics of obesity and diabetes [10-22]. National food, nutrition, and health policies and programs have positioned low-fat milk as the preferred caloric replacement strategy for SSBs [23-27]. This strategy derives from evidence that replacement of SSBs with low-fat milk is associated with reductions in weight and incident diabetes in prospective cohort studies [28, 29] and reduces liver fat (an important early metabolic lesion linking obesity to diabetes), as well as triglycerides and blood pressure in randomized trials [30]. Whether these benefits hold for soy milk alternatives, which have shown distinct advantages over cow's milk [7, 31-33], is unclear. There is an urgent need for studies to clarify the benefits of soy milk as an alternative to cow's milk.

3. OBJECTIVES, ENDPOINTS AND HYPOTHESES

Table 1. Objectives, endpoints and hypotheses

OBJECTIVE	ENDPOINT	HYPOTHESIS
**PRIMARY**		
To assess the effect of using 2% soy milk versus 2% cow's milk to replace SSBs on liver fat in overweight/obese participants with MetS.	Liver fat (intrahepatocellular lipid [IHCL])	• 2% soy milk and 2% cow's milk intake will each result in a significant liver fat reduction compared to SSBs intake in overweight/obese participants with MetS.<br>• 2% soy milk intake will show to be non-inferior to 2% cow's milk intake on liver fat reduction in overweight/obese participants with MetS.
**SECONDARY**		
To assess the effect of using 2% soy milk versus 2% cow's milk to replace SSBs on insulin sensitivity in overweight/obese participants with MetS.	Whole body insulin sensitivity (Matsuda insulin sensitivity index [ISI] [34])	• 2% soy milk and 2% cow's milk intake will each improve insulin sensitivity compared to SSBs intake in overweight/obese participants with MetS.<br>• 2% soy milk intake will show to be non-inferior to 2% cow's milk intake on insulin sensitivity in overweight/obese participants with MetS.

To assess the effect of using 2% soy milk versus 2% cow's milk to replace SSBs on beta-cell function in overweight/obese participants with MetS.	Beta-cell function (insulin secretion-sensitivity index-2 [ISSI-2][35, 36])	• 2% soy milk and 2% cow's milk intake will each improve beta-cell function compared to SSBs intake in overweight/obese participants with MetS.<br>• 2% soy milk intake will show to be non-inferior to 2% cow's milk intake on beta-cell function in overweight/obese participants with MetS.
To assess the effect of using 2% soy milk versus 2% cow's milk to replace SSBs on glucose tolerance in overweight/obese participants with MetS.	Glucose tolerance (area under the curve [AUC])	• 2% soy milk and 2% cow's milk intake will each improve glucose tolerance compared to SSBs intake in overweight/obese participants with MetS.<br>• 2% soy milk intake will show to be non-inferior to 2% cow's milk intake on glucose tolerance in overweight/obese participants with MetS.
To assess the effect of using 2% soy milk versus 2% cow's milk to replace SSBs on glucose tolerance in overweight/obese participants with MetS.	Glucose tolerance (2h plasma glucose [2h-PG])	• 2% soy milk and 2% cow's milk intake will each improve glucose tolerance compared to SSBs intake in overweight/obese participants with MetS.<br>• 2% soy milk intake will show to be non-inferior to 2% cow's milk intake on glucose tolerance in overweight/obese participants with MetS.
**OTHER PRE-SPECIFIED OUTCOME MEASURES**		
To assess the effect of using 2% soy milk versus 2% cow's milk to replace SSBs on ectopic muscle fat in overweight/obese participants with MetS.	Ectopic muscle fat (intramyocellular lipid [IMCL])	• 2% soy milk and 2% cow's milk intake will each result in a significant ectopic muscle fat reduction compared to SSBs intake in overweight/obese participants with MetS.<br>• 2% soy milk intake will show to be non-inferior to 2% cow's milk intake on ectopic muscle fat reduction in overweight/obese participants with MetS.

To assess the effect of using 2% soy milk versus 2% cow's milk to replace SSBs on waist circumference in overweight/obese participants with MetS.	MetS criteria (waist circumference,)	• 2% soy milk and 2% cow's milk intake will each result in significant waist circumference reduction compared to SSBs intake in overweight/obese participants with MetS.<br>• 2% soy milk intake will show to be non-inferior to 2% cow's milk intake on waist circumference reduction in overweight/obese participants with MetS.
To assess the effect of using 2% soy milk versus 2% cow's milk to replace SSBs on fasting plasma glucose in overweight/obese participants with MetS.	MetS criteria (fasting plasma glucose)	• 2% soy milk and 2% cow's milk intake will each result in significant fasting plasma glucose reduction compared to SSBs intake in overweight/obese participants with MetS.<br>• 2% soy milk intake will show to be non-inferior to 2% cow's milk intake on fasting plasma glucose reduction in overweight/obese participants with MetS.
To assess the effect of using 2% soy milk versus 2% cow's milk to replace SSBs on fasting serum triglycerides in overweight/obese participants with MetS.	MetS criteria (fasting serum triglycerides)	• 2% soy milk and 2% cow's milk intake will each result in significant fasting serum triglycerides reduction compared to SSBs intake in overweight/obese participants with MetS.<br>• 2% soy milk intake will show to be non-inferior to 2% cow's milk intake on fasting serum triglycerides reduction in overweight/obese participants with MetS.
To assess the effect of using 2% soy milk versus 2% cow's milk to replace SSBs on fasting serum HDL-C in overweight/obese participants with MetS.	MetS criteria (fasting serum HDL-C)	• 2% soy milk and 2% cow's milk intake will each result in significant fasting serum HDL-C increase compared to SSBs intake in overweight/obese participants with MetS.<br>• 2% soy milk intake will show to be non-inferior to 2% cow's milk intake on fasting serum HDL-C increase in overweight/obese participants with MetS.

To assess the effect of using 2% soy milk versus 2% cow's milk to replace SSBs on SBP in overweight/obese participants with MetS.	MetS criteria (SBP)	• 2% soy milk and 2% cow's milk intake will each result in significant SBP reduction compared to SSBs intake in overweight/obese participants with MetS.<br>• 2% soy milk intake will show to be non-inferior to 2% cow's milk intake on SBP reduction in overweight/obese participants with MetS.
To assess the effect of using 2% soy milk versus 2% cow's milk to replace SSBs on DBP in overweight/obese participants with MetS.	MetS criteria (DBP)	• 2% soy milk and 2% cow's milk intake will each result in significant DBP reduction compared to SSBs intake in overweight/obese participants with MetS.<br>• 2% soy milk intake will show to be non-inferior to 2% cow's milk intake on DBP reduction in overweight/obese participants with MetS.
To assess the effect of using 2% soy milk versus 2% cow's milk to replace SSBs on MetS reversion in overweight/obese participants with MetS.	MetS reversion (defined as a reversion to <3 of 5 IDF/NHLBI/AHA/WHF/IAAS/IASO criteria [37])	• 2% soy milk and 2% cow's milk intake will each result in significant MetS reversion compared to SSBs intake in overweight/obese participants with MetS.<br>• 2% soy milk intake will show to be non-inferior to 2% cow's milk intake on MetS reversion in overweight/obese participants with MetS.
To assess the effect of using 2% soy milk versus 2% cow's milk to replace SSBs on diabetes incidence in overweight/obese participants with MetS.	Diabetes Incidence (Diabetes Canada 2018 FPG and 2h-PG [75g-OGTT] or HbA1c criteria)[38]	• 2% soy milk and 2% cow's milk intake will each result in significant lower diabetes incidence compared to SSBs intake in overweight/obese participants with MetS.<br>• 2% soy milk intake will show to be non-inferior to 2% cow's milk intake on diabetes incidence in overweight/obese participants with MetS.

To assess the effect of using 2% soy milk versus 2% cow's milk to replace SSBs on body weight in overweight/obese participants with MetS.	Body weight	• 2% soy milk and 2% cow's milk intake will each result in significant body weight reduction compared to SSBs intake in overweight/obese participants with MetS.<br>• 2% soy milk intake will show to be non-inferior to 2% cow's milk intake on body weight reduction in overweight/obese participants with MetS.
To assess the effect of using 2% soy milk versus 2% cow's milk to replace SSBs on gut microbiome diversity in overweight/obese participants with MetS.	Microbiome diversity (16S rRNA sequencing)	• 2% soy milk and 2% cow's milk intake will each result in significant increase in gut microbiome diversity compared to SSBs intake in overweight/obese participants with MetS.<br>• 2% soy milk intake will show to be non-inferior to cow's milk intake on increase in gut microbiome diversity in overweight/obese participants with MetS.
To assess the effect of using 2% soy milk versus 2% cow's milk to replace SSBs on NAFLD markers (alanine transaminase [ALT]) in overweight/obese participants with MetS.	NAFLD markers (ALT)	• 2% soy milk and 2% cow's milk intake will each result in significant ALT reduction compared to SSBs intake in overweight/obese participants with MetS.<br>• 2% soy milk intake will show to be non-inferior to 2% cow's milk intake on ALT reduction in overweight/obese participants with MetS.
To assess the effect of using 2% soy milk versus 2% cow's milk to replace SSBs on NAFLD markers (aspartate transaminase [AST]) in overweight/obese participants with MetS.	NAFLD markers (AST)	• 2% soy milk and 2% cow's milk intake will each result in significant AST reduction compared to SSBs intake in overweight/obese participants with MetS.<br>• 2% soy milk intake will show to be non-inferior to 2% cow's milk intake on AST in overweight/obese participants with MetS.

To assess the effect of using 2% soy milk versus 2% cow's milk to replace SSBs on NAFLD markers (gamma-glutamyl transferase [GGT]) in overweight/obese participants with MetS.	NAFLD markers (GGT)	• 2% soy milk and 2% cow's milk intake will each result in significant GGT reduction compared to SSBs intake in overweight/obese participants with MetS.<br>• 2% soy milk intake will show to be non-inferior to 2% cow's milk intake on GGT reduction in overweight/obese participants with MetS.
To assess the effect of using 2% soy milk versus 2% cow's milk to replace SSBs on NAFLD markers (alkaline phosphatase [ALP]) in overweight/obese participants with MetS.	NAFLD makers (ALP)	• 2% soy milk and 2% cow's milk intake will each result in significant ALP reduction compared to SSBs intake in overweight/obese participants with MetS.<br>• 2% soy milk intake will show to be non-inferior to 2% cow's milk intake on ALP reduction in overweight/obese participants with MetS.
To assess the effect of using 2% soy milk versus 2% cow's milk to replace SSBs on NAFLD markers (fatty liver index [FLI]) in overweight/obese participants with MetS.	NAFLD markers (FLI) [39]	• 2% soy milk and 2% cow's milk intake will each result in significant FLI reduction compared to SSBs intake in overweight/obese participants with MetS.<br>• 2% soy milk intake will show to be non-inferior to 2% cow's milk intake on FLI reduction in overweight/obese participants with MetS.
To assess the effect of using 2% soy milk versus 2% cow's milk to replace SSBs on hepatic insulin resistance (HOMA-IR) in overweight/obese participants with MetS.	Hepatic insulin resistance (HOMA-IR)	• 2% soy milk and 2% cow's milk intake will each result in significant insulin resistance reduction compared to SSBs intake in overweight/obese participants with MetS.<br>• 2% soy milk intake will show to be non-inferior to 2% cow's milk intake on insulin resistance reduction in overweight/obese participants with MetS.

To assess the effect of using 2% soy milk versus 2% cow's milk to replace SSBs on uric acid in overweight/obese participants with MetS.	Uric acid	• 2% soy milk and 2% cow's milk intake will each result in significant uric acid reduction compared to SSBs intake in overweight/obese participants with MetS.<br>• 2% soy milk intake will show to be non-inferior to 2% cow's milk intake on uric acid reduction in overweight/obese participants with MetS.
To assess the effect of using 2% soy milk versus 2% cow's milk to replace SSBs on established lipid targets (LDL-C) in overweight/obese participants with MetS.	Established lipid targets – LDL-C	• 2% soy milk and 2% cow's milk intake will each result in significant established lipid targets (LDL-C) reduction compared to SSBs intake in overweight/obese participants with MetS.<br>• 2% soy milk intake will show to be non-inferior to 2% cow's milk intake on established lipid targets (LDL-C) reduction in overweight/obese participants with MetS.
To assess the effect of using 2% soy milk versus 2% cow's milk to replace SSBs on established lipid targets (non-HDL-C) in overweight/obese participants with MetS.	Established lipid targets – non-HDL-C	• 2% soy milk and 2% cow's milk intake will each result in significant established lipid targets (non-HDL-C) reduction compared to SSBs intake in overweight/obese participants with MetS.<br>• 2% soy milk intake will show to be non-inferior to 2% cow's milk intake on established lipid targets (non-HDL-C) reduction in overweight/obese participants with MetS.

To assess the effect of using 2% soy milk versus 2% cow's milk to replace SSBs on established lipid targets (Total Cholesterol) in overweight/obese participants with MetS.	Established lipid targets- Total Cholesterol	• 2% soy milk and 2% cow's milk intake will each result in significant established lipid targets (Total Cholesterol) reduction compared to SSBs intake in overweight/obese participants with MetS.<br>• 2% soy milk intake will show to be non-inferior to 2% cow's milk intake on established lipid targets (Total Cholesterol) reduction in overweight/obese participants with MetS.
To assess the effect of using 2% soy milk versus 2% cow's milk to replace SSBs on inflammation in overweight/obese participants with MetS.	Inflammation (high-sensitivity C-reactive protein [hs-CRP])	• 2% soy milk and 2% cow's milk intake will each result in significant inflammation reduction compared to SSBs intake in overweight/obese participants with MetS.<br>• 2% soy milk intake will show to be non-inferior to 2% cow's milk intake on inflammation reduction in overweight/obese participants with MetS.
To assess the effect of using 2% soy milk versus 2% cow's milk to replace SSBs on kidney function/injury (creatinine) in overweight/obese participants with MetS.	Kidney function/injury – serum Creatinine	• 2% soy milk and 2% cow's milk intake will each result in significant kidney function/injury (creatinine) reduction compared to SSBs intake in overweight/obese participants with MetS.<br>• 2% soy milk intake will show to be non-inferior to 2% cow's milk intake on kidney function/injury (creatinine) reduction in overweight/obese participants with MetS.

To assess the effect of using 2% soy milk versus 2% cow's milk to replace SSBs on kidney function/injury (eGFR) in overweight/obese participants with MetS.	Kidney function/injury - eGFR	• 2% soy milk and 2% cow's milk intake will each result in significant kidney function/injury (e-GFR) reduction compared to SSBs intake in overweight/obese participants with MetS.<br>• 2% soy milk intake will show to be non-inferior to 2% cow's milk intake on kidney function/injury (eGFR) reduction in overweight/obese participants with MetS.
To assess the effect of using 2% soy milk versus 2% cow's milk to replace SSBs on kidney function/injury (urinary albumin excretion rate [AER]) in overweight/obese participants with MetS.	Kidney function/injury – urinary AER	• 2% soy milk and 2% cow's milk intake will each result in significant kidney function/injury (AER) reduction compared to SSBs intake in overweight/obese participants with MetS.<br>• 2% soy milk intake will show to be non-inferior to 2% cow's milk intake on kidney function/injury (AER) reduction in overweight/obese participants with MetS.
To assess the effect of using 2% soy milk versus 2% cow's milk to replace SSBs on kidney function/injury (urinary albumin/creatinine ratio [ACR]) in overweight/obese participants with MetS.	Kidney function/injury – urinary ACR	• 2% soy milk and 2% cow's milk intake will each result in significant kidney function/injury (ACR) reduction compared to SSBs intake in overweight/obese participants with MetS.<br>• 2% soy milk intake will show to be non-inferior to 2% cow's milk intake on kidney function/injury (ACR) reduction in overweight/obese participants with MetS.

To assess the effect of using 2% soy milk versus 2% cow's milk to replace SSBs on diet quality in overweight/obese participants with MetS.	Diet quality (Alternative Healthy Eating Index [AHEI] using the Harvard Willett food frequency questionnaire [FFQ] [40] and the Healthy Eating Index [HEI] using the Diet ID^{TM} online platform [41]	• 2% soy milk and 2% cow's milk intake will each result in significant higher diet quality score compared to SSBs intake in overweight/obese participants with MetS.<br>• 2% soy milk intake will show to be non-inferior to 2% cow's milk intake on diet quality score in overweight/obese participants with MetS.
To assess the effect of using 2% soy milk versus 2% cow's milk to replace SSBs on appetite in overweight/obese participants with MetS.	Appetite	• 2% soy milk and 2% cow's milk intake will each result in significant appetite reduction compared to SSBs intake in overweight/obese participants with MetS.<br>• 2% soy milk intake will show to be non-inferior to 2% cow's milk intake on appetite reduction in overweight/obese participants with MetS.
To assess the effect of using 2% soy milk versus 2% cow's milk to replace SSBs on food craving control in overweight/obese participants with MetS.	Food craving control	• 2% soy milk and 2% cow's milk intake will each result in significant food craving control increase compared to SSBs intake in overweight/obese participants with MetS.<br>• 2% soy milk intake will show to be non-inferior to 2% cow's milk intake on food craving control increase in overweight/obese participants with MetS.
To assess the effect of using 2% soy milk versus 2% cow's milk to replace SSBs on food craving for savoury in overweight/obese participants with MetS.	Food craving for savoury	• 2% soy milk and 2% cow's milk intake will each result in significant food craving for savoury reduction compared to SSBs intake in overweight/obese participants with MetS.<br>• 2% soy milk intake will show to be non-inferior to 2% cow's milk intake on food craving for savoury reduction in overweight/obese participants with MetS.

To assess the effect of using 2% soy milk versus 2% cow's milk to replace SSBs on food cravings for sweet in overweight/obese participants with MetS.	Food cravings for sweet	• 2% soy milk and 2% cow's milk intake will each result in significant food cravings for sweet reduction compared to SSBs intake in overweight/obese participants with MetS.<br>• 2% soy milk intake will show to be non-inferior to 2% cow's milk intake on food cravings for sweet reduction in overweight/obese participants with MetS.
To assess the effect of using 2% soy milk versus 2% cow's milk to replace SSBs on perceived craving control over SSBs in overweight/obese participants with MetS.	Perceived craving control over SSBs	• 2% soy milk and 2% cow's milk intake will each result in significant perceived craving control (over SSBs) increase compared to SSBs intake in overweight/obese participants with MetS.<br>• 2% soy milk intake will show to be non-inferior to 2% cow's milk intake on perceived craving control (over SSBs) increase in overweight/obese participants with MetS.
To assess the effect of using 2% soy milk versus 2% cow's milk to replace SSBs on positive mood in overweight/obese participants with MetS	Positive mood	• 2% soy milk and 2% cow's milk intake will each result in significant positive mood increase compared to SSBs intake in overweight/obese participants with MetS.<br>• 2% soy milk intake will show to be non-inferior to 2% cow's milk intake on positive mood increase in overweight/obese participants with MetS.

To assess the adherence to 2% soy milk intake, 2% cow's milk intake and SSBs intake during the 24-week treatment period using study beverage logs at weeks 0, 4, 8, 12, 16, 20 and 24, and using objective biomarkers at weeks 0, 12 and 24.	Study beverage adherence:<br>• % of study beverage adherence (using study beverage logs)<br>• Change in objective biomarkers:<br>1. 2% soy milk intake (urinary isoflavonones excretion [UIE] [42]<br>2. 2% cow's milk intake (dairy-derived serum fatty acids [15:0, 17:0, conjugated linoleic acid [CLA], trans-palmitoleic acid [TPA] [43]<br>3. SSBs intake (Urinary fructose and sucrose)	• About 60% of participants will adhere to the study beverage intake during the 24-week treatment period.<br>• There will be an increase/change in all objective biomarkers from week 0 to week 12 and week 0 to week 24.

4. STUDY DESIGN

4.1. Overall Design

The trial will be conducted at the MRI Research Unit and at the Clinical Nutrition and Risk Factor Modification Centre at St. Michael's Hospital. The trial will be a 24-week, 2-phase, 3-arm, non-inferiority, open-label, parallel group, randomized controlled trial to assess the effect of replacing SSBs with 2% soy milk versus 2% cow's milk on the primary outcome liver fat and secondary outcomes muscle fat, insulin sensitivity, beta-cell function, glucose tolerance and established cardiometabolic risk factors in overweight/obese participants who are consuming ≥1 SSBs/day (see Figure 1). The trial will be conducted according to Good Clinical Practice and reported according to CONSORT and the COSORT extension for non-inferiority trials [44].

All participants will be expected to participate in the study for 28 weeks (one run-in phase of four weeks, and 24-week intervention period) and be able to attend all study visits. After randomization, all study beverages will be provided with the instruction to replace usual SSBs intake while maintaining background diets.

SSBs will be defined as any sugar-sweetened beverage (sodas and soft drinks) that contains at least 50 kcal per 8-oz serving. For the purpose of the study, SSBs will include sports/energy drinks, sweetened iced tea, homemade SSBs such as frescas or fruit drinks, but will NOT include coffee or 100% fruit juice.

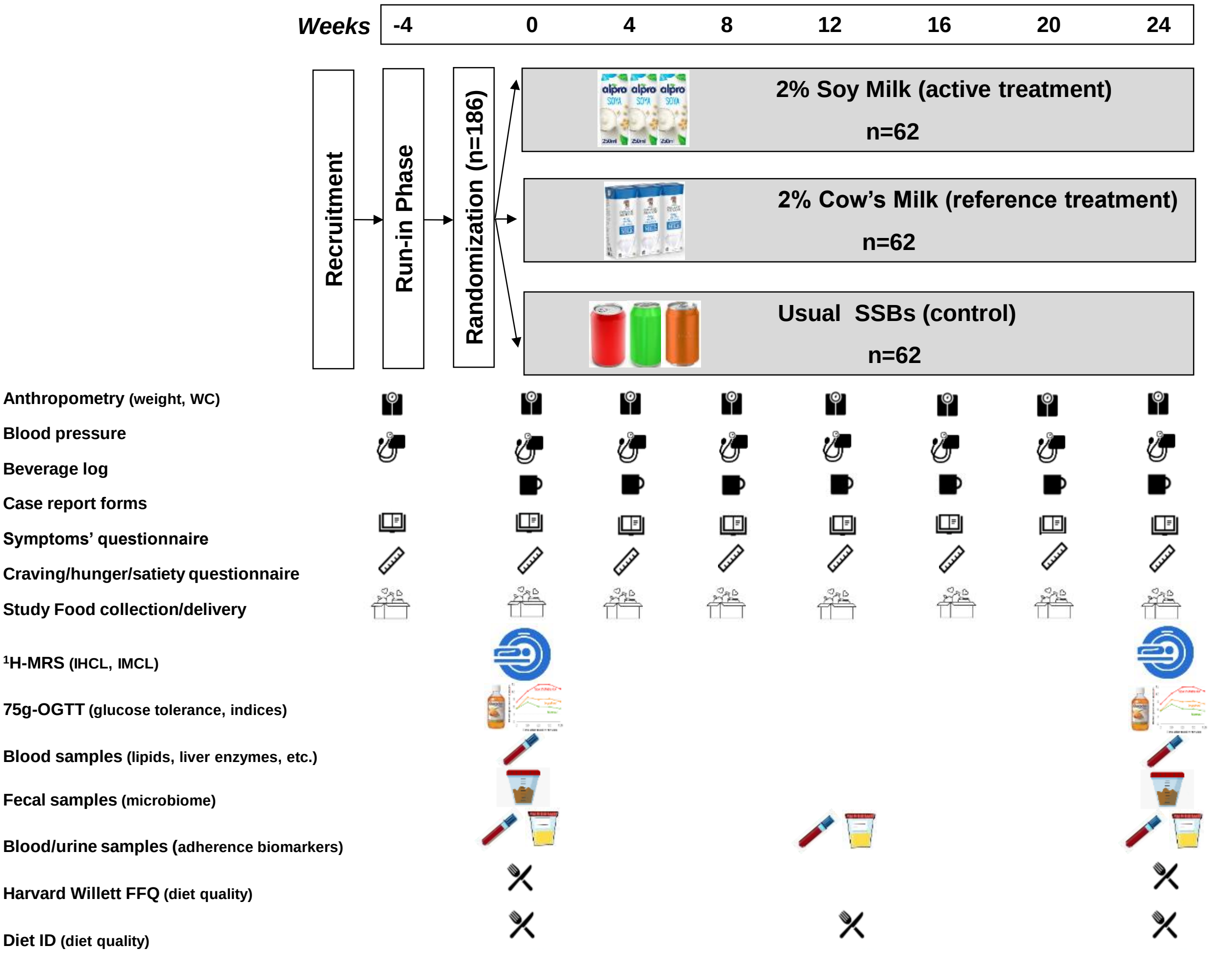

Figure 1. Design of the Soy Treatment Evaluation on the progression of Metabolic health (STEM) trial

4.2. Scientific Rationale for study design

The 3-arm design has the advantage of combing a superiority trial with a non-inferiority trial, by allowing us to test the superiority of the active (2% soy milk) and reference (2% cow's milk) treatments over the control (SSBs) and the noninferiority of the active (2% soy milk) compared with the reference (2% cow's milk) treatment simultaneously. The demonstration that soy milk is non-inferior or even superior to cow's milk (that is, "as good as" or better than cow's milk) as a replacement strategy for SSBs would be an actionable outcome for soy as it would position soy as a healthy alternative to cow's milk for SSBs reduction policies and programs with important implications for food procurement policies at public institutions which currently recommend that SSBs be replaced with low-fat milk in vending machines and food service operations at schools, hospitals, and government agencies in the U.S., Canada, and Europe [23-27]. We chose a 4-week run-in phase as a strategy to enhance retention and a treatment duration of 24-weeks as this was the duration over which milk was shown to reduce liver fat in

replacement for SSBs [30] and is a duration that will have a meaningful impact on public health policy [45, 46].

The anticipated outcomes will address other important questions. The demonstration that 2% soy milk is at least non-inferior or even superior to 2% cow's milk (that is, "as good as" or better than cow's milk) will leverage and reinforce the 2020 Dietary Guidelines for Americans (DGA) messages that soy milk is the only plant-based milk considered to be a dairy equivalent that can contribute to meeting dairy recommendations [47]. The anticipated outcomes will also directly challenge the notion that soy milk and plant-based soy foods as ultra-processed foods are unhealthy. By the WHO/FAO-endorsed NOVA classification system [48], soy milk is ultra-processed, while cow's milk is unprocessed or minimally processed and SSBs are ultra-processed. The proposed trial will allow us to test whether an ultra-processed food (soy milk) can be "as good as" or better than a matched unprocessed or minimally processed food (cow's milk). Taken together with the results of the SWAP-MEAT trial [49], our results will demonstrate the failure of the NOVA classification system when applied to plant-based soy foods. This finding could have important implications for fiscal policy/market interventions (taxes and subsidies) and statutory regulations (sales and advertising bans/restrictions) that have already been recommended against ultra-processed foods by WHO/PAHO [50].

4.3. Sample Size (Power) Calculation

A total of 186 participants will be randomized. N=62 per group will allow us to test the non-inferiority of the active (2% soy milk) compared with the reference (2% cow's milk) treatment with a non-inferiority margin (δ) of 75% of the effect of the reference treatment on the primary outcome intra-hepatocellular lipid (IHCL) by ^{1}H-MRS (1.5% absolute difference in IHCL based on a 75% preserved fraction of an expected 2% absolute difference in IHCL) by difference of means[51], assuming an SD of 3%, α=0.05, 80% power (β=0.20), and 20% attrition. A 2% absolute difference in IHCL was chosen as it is the minimum difference associated with clinically meaningful changes in downstream glycemic control, insulin sensitivity, blood pressure, and/or blood lipids [52-57]. A non-inferiority margin (δ) of 75% was selected based on guidance from the Food and Drug Administration (FDA) and European Medicines Agency [58-60]. This sample size will provide us ample power to test the superiority of the active (2% soy milk) and the reference (2% cow's milk) treatments compared with the control (SSBs) treatment assuming the same 2% absolute difference in IHCL, SD of 3%, α=0.05, 91% power (β=0.20), and 20% attrition. It will also provide us with sufficient power for superiority testing of the secondary outcomes.

4.4. Recruitment

We will use a digital marketing groups, Trialfacts or Honeybee, to launch a campaign to recruit participants. Additional recruitment will include internal list, advertising on social media (Facebook and Instagram), advertisement in newspapers, online listings on Craigslist and Kijiji, and postings and flyer handouts at St. Michael's Hospital, the University of Toronto, local clinics, and surrounding areas (including grocery stores, community/recreation centres, convenience stores, malls, drug stores, places

of worship, public message boards). Trialfacts and Honeybee work exclusively with research groups to provide specialized patient recruitment services for clinical trials. We will be retaining data of screen failures for CONSORT statement purposes. Eligible participants will be contacted via phone or email as they indicate in the completion of the Trialfacts or Honeybee surveys, internal list or direct mail service from Canada Post. All potential participants will be invited to attend a virtual information session on the study. Based on our most recent Stop Sugars NOW trial (NCT03543644) with similar recruitment criteria, we estimate a recruitment rate of 6-8 participants/week and therefore plan to recruit all the participants within a 6-month period. The research coordinators will organize the recruitment, the screening and consent.

4.5. Screening and Consent

After the information session, interested individuals will be contacted and screened over the phone for eligibility using a questionnaire and be briefed on the nature of the study, treatments, and procedures. Individuals who qualify and are interested will be booked for an additional in-person screening and if consented to provide an email address, we will send the study consent form in advance so they have ample time to review study procedures. Based on our experience, we will need to consent 500 participants.

Participants who provide consent will be assigned a screening identification (ID) and will be scheduled for an in-person screening at the Clinical Nutrition and Risk Factor Modification Centre at the St. Michael's Hospital (study site). During the in-person screening, participants will be instructed on details of the trial protocol and after signing the study consent, will have their anthropometric measures (height, weight, waist circumference, and blood pressure) taken. The participants will receive study treatment beverage samples to assess if they are able to consume the products for the six months, if randomized to either of the intervention groups.

Those who meet all the eligibility criteria will be enrolled in the run-in phase of the study, which will last four weeks, and then be assigned a study ID on the day they are randomized to one of the three arms of the study and begin the 24-week intervention treatment.

5. STUDY POPULATION

5.1. Inclusion Criteria

In order to be eligible to participate in this study, an individual must meet all of the following criteria:

- Adults (age 18-75 years), men and non-pregnant women
- Overweight or obese (BMI ≥25kg/m^2)
- High waist circumference (USA/Canada ≥102cm in men, ≥88cm in women; Europid/Caucasian/Middle East, Mediterranean/Sub-Saharan African ≥94cm in men, ≥80cm in

women; and Asian [including Japanese]/Ethnic Central and South American ≥90cm in men, ≥80cm in women[37], with a waist diameter ≤60 cm)
- Regularly drinking SSBs (≥ 1 servings/day)

We will include participants consuming ≥ 1 SSBs servings/day, with 50% enrichment of those consuming ≥ 3 SSBs servings/day.

Participants must be agreeable to consume only one study beverage for the duration of the intervention. Participants that are randomized to the 2% soy milk group will be asked to substitute their regular SSB consumption with the 2% soy milk (up to a max of 6 servings/day). Similarly, participants that are randomized to the 2% cow's milk group will be asked to substitute their regular SSB consumption with the 2% cow's milk. All participants must be proficient in English to be eligible for the study. Please see exclusion criteria for a full set of exclusion criteria.

Participants must consent to have the study drinks shipped to their home address by a third-party company, who will not be informed of their participation in the study, or be agreeable to pick up study drinks from the study site at St. Michael's Hospital.

5.2. Exclusion Criteria

An individual who meets any of the following criteria will be excluded from participation in this study.

- Age <18 or >75 years.
- BMI <25.
- Waist circumference lower than threshold[37] (USA/Canada <102cm in men, <88cm in women; Europid/Caucasian/Middle East, Mediterranean/Sub-Saharan African <94cm in men, <80cm in women; and Asian [including Japanese]/Ethnic Central and South American <90cm in men, <80cm in women) or a waist diameter >60cm.
- Uncontrolled hypertension (or systolic blood pressure ≥ 180 mmHg or diastolic ≥ 110 mmHg
- Self-reported diabetes
- Not regularly drinking SSBs (<1 serving per day)
- Self-reported cow's milk or soy intolerance or allergy
- Self-reported pregnant or breast-feeding females, or women planning on becoming pregnant throughout the study period
- Self-reported weight loss of ≥10% in the last 6 months
- Complementary or alternative medicine (CAM) use as deemed inappropriate by investigators
- Self-reported Wilson's disease
- Self-reported haemochromatosis
- Self-reported inborn errors of metabolism

- Self-reported lipodystrophy
- Self-reported Cushing syndrome or disease
- Self-reported gastrointestinal disease (inflammatory bowel disease or malabsorption disorder)
- Previous bariatric surgery
- Self-reported alcoholic fatty liver disease, cirrhosis, hepatocellular carcinoma, HCV, HBV, or HAV infection, or genetic causes of liver disease (Alpha-1-antitrypsin [A1A] deficiency)
- Self-reported uncontrolled hyperthyroidism or hypothyroidism
- Self-reported high risk or very high risk chronic kidney disease (CKD) (KIDIGO 2012 criteria)
- Self-reported acute or chronic infection (e.g. salmonellosis, HIV, TB)
- Self-reported chronic inflammatory conditions
- Self-reported chronic lung disease
- Self-reported chronic pancreatitis or pancreatic insufficiency
- Self-reported cystic fibrosis
- Self-reported cancer/malignancy in the last 6 months, with the exception of skin cancer
- Self-reported schizophrenia spectrum and other psychotic disorders, bipolar and related disorders, and dissociative disorders
- Self-reported severe depression
- Self-reported major surgery in the last 6 months
- Self-reported hypopituitarism
- Self-reported hypogonadism
- Self-reported substance abuse disorder (substance dependence including alcohol or recreational drugs)
- Participation in any trials within the last 3 months or for the duration of this study
- Any condition or circumstance which would prevent you from having an MRI (e.g. pacemaker, neurostimulators, breast tissue expanders, implants, or foreign metal object in body)
- Individuals planning on making dietary or physical activity changes throughout study duration
- If self-reported medication use, it must be at a stable dose for ≥6 months.

*Disease exclusions will be based upon self-reported diagnosis

5.3. Lifestyle Considerations

During the duration of the study, participants are asked to:

Refrain from starting medications or dietary supplements and to continue doing the same or similar physical activity

6. STUDY INTERVENTIONS

6.1. Interventions

The 3 interventions will consist of ≥1 servings of (1) 2% soy milk (Alpro Soya[SBI], 250mL single-serve shelf-stable packs, 100Kcal with 8g soy protein per 250mL), (2) 2% cow's milk (Organic Meadows®,

250mL, single-serve shelf-stable packs, 130Kcal with 8g casein/whey protein per 250mL), or (3) usual SSBs (355mL single-serve cans, 130-140kcal per 355mL). The participant's baseline level of intake of SSBs will determine the dose (number of servings). The advantage of this approach is that it represents a 'real-world', 'one-for-one' substitution and allows for a natural dose response. We will cap the maximum allowable number of servings at 6 servings/day, to avoid extreme intakes and ensure that the intake of soy isoflavones does not exceed the generally recognized upper limit of safe intake of 150 mg/day[61, 62]. Although the soy and milk interventions will be matched for protein and serving size, we intentionally did not make the 3 interventions isocaloric, as the goal was to use "real-world" product substitutions. Participants will be instructed to replace their usual SSBs intake with the study beverages while freely consuming their usual background diets. All intervention beverages will be provided. Participants will only receive the beverages that correspond to their group assignment. They will pick up one week of their assigned beverages at each study visit and then will have the remaining three weeks of beverages delivered using an online delivery service (Starhawk/Instacart). Research coordinators will coordinate the beverage disbursements.

Table 3. Intervention beverages.

2% Soy milk group	2% Cow's milk group (Contains lactose)	SSBs group
Alpro Soya<br><br>250 mL, single-serve shelf-stable pack<br><br>100 Kcal, 8g soy protein	Organic Meadows®<br><br>250 mL, single-serve shelf-stable pack<br><br>130 Kcal, 8g casein/whey protein	355 mL, single-serve cans<br><br>130-140 Kcal, 0g protein

6.2. Measures to minimize bias: randomization and blinding

6.2.1. Randomization

The Applied Health Research Centre (AHRC) will perform block randomization with allocation concealment through the Research Electronic Data Capture (REDCap) program. Following successful completion of the run-in phase, participants will be randomized into 3 groups from each strata, using random permuted blocks with unequal sizes.

6.2.2. Blinding

Blinding of the participants and investigators will not be possible due to packaging, taste and look of the interventions. However, outcome assessors (laboratory, microbiome analysis) and the statistician will be blinded to the identity of the treatments.

6.3. Study visits

The trial will be conducted at the CFI-funded MRI Research Unit and at the Clinical Nutrition and Risk Factor Modification Centre at St. Michael's Hospital.

6.3.1. Run-in Phase

Participants who are eligible will start the 4-week run-in phase. For four weeks during the run-in phase and prior to the first study visit, participants will begin to log their usual beverage intake as well as asked for their beverage preference of SSB. They will fill out the medication and supplement list, medical history form, physical activity questionnaire, symptoms checklist and control eating questionnaire. They will also be instructed on how to collect a 24-hour urine sample and fecal sample and provided kits to do so for the first study visit.

6.3.2. Study visits (Weeks 0, 4, 8, 12, 16, 20 and 24, a total of 7 study visits)

At each visit, study personnel will do anthropometric measurements (weight and waist circumference), take their blood pressure, collect the beverage logs and provide new beverage logs. They will also do case report forms, medication and supplement list, medical history form, physical activity questionnaire, symptoms checklist, control eating questionnaire and provide participants with the study beverages. Participants will receive motivational phone calls every 2-weeks between visits to provide reminders and ensure fidelity to the protocol.

At weeks 0, 12 and 24, adherence biomarkers in blood and urine samples will be collected and participants will do the diet quality assessment (Diet ID). Study personnel will obtain the 24-hour urine collection from participants at the study visits. A registered nurse will collect the blood sample, at weeks 0 and 24 at the time of the OGTT blood collection, and at week 12, the blood sample collection will be done by a single venipuncture. Participants will be given a unique user and visit code (for weeks 0,12 and 24) number for logging in to the Diet ID [41] website.

At weeks 0 and 24, participants will have the following assessed/collected: ^{1}H-MRS (IHCL, IMCL), the 75g-OGTT (glucose tolerance, indices) [63], blood samples (lipids, liver enzymes, etc), fecal samples (microbiome), and diet quality (Harvard Willett FFQ). The ^{1}H-MRS will be performed to measure ectopic fat in liver and calf muscles. Participants will have the ^{1}H-MRS at the CFI-funded MRI Research Unit at St. Michael's Hospital and will continue their visit at the Clinical Nutrition and Risk Factor Modification Centre located also at St. Michael's Hospital. Participants will come in to the study site in a 10 to 12-hour fasted state. They will be instructed to maintain the same dietary and exercise patterns the evening before each test and to consume a minimum of 150g of carbohydrate each day over the three days prior to the test. To ensure that these instructions are followed, participants will be provided with examples of what constitutes 150g of carbohydrate. A fasting blood sample for secondary, exploratory and adherence outcomes (including glucose, insulin, lipid panel, ALT and other liver enzymes, and other

markers of glucose control, biomarkers of adherence, and other biomarkers of cardiometabolic risk) will be taken by a registered nurse. A standard protocol will be followed for the administration of a 2-hour 75g-OGTT. Participants will have a catheter inserted into a forearm vein by the nurse, secured by tape, and kept patent by saline. Two baseline samples will be obtained in the fasting state at -30 and 0-min. A 75g OGTT meal (GlucoDex 75g, Rougier Pharma Mirabel, QC, Canada) will then be administered with instructions to consume it over exactly 5 min at a constant rate using a timer. Additional blood samples will be drawn at 30, 60, 90, and 120 min after the start of the test, at the end of the test the nurse will remove the catheter. Breakfast will be prepared at the study site kitchen and provided to the participants after the OGTT.

Table 4: Participant Visit Schedule

Measurements	Screening	Run-in (Week -4)	Week 0	Week 4	Week 8	Week 12	Week 16	Week 20	Week 24
**Study Questionnaires**									
Contact information	X								
Medications and supplements	X	X	X	X	X	X	X	X	X
Medical history	X	X	X	X	X	X	X	X	X
Physical activity	X	X	X	X	X	X	X	X	X
Beverage log			X	X	X	X	X	X	X
Case report forms	X	X	X	X	X	X	X	X	X
Symptoms' questionnaire		X	X	X	X	X	X	X	X
Craving/hunger/satiety questionnaire		X	X	X	X	X	X	X	X
Study Food collection/delivery		X	X	X	X	X	X	X	X
Diet ID (diet quality)			X			X			X
Harvard Willett FFQ (diet quality)			X						X
**Anthropometric Measures and Blood Pressure**									
Height	X								
Weight	X	X	X	X	X	X	X	X	X
Waist circumference	X	X	X	X	X	X	X	X	X
Blood pressure	X	X	X	X	X	X	X	X	X
**Biochemical Measures**									
75g-OGTT (glucose tolerance, indices)			X						X
Blood samples (lipids, liver enzymes, etc)			X						X
Fecal samples (microbiome)			X						X
Blood and urine samples (adherence biomarkers)			X			X			X
**Medical Imaging**									
^{1}H-MRS (IHCL, IMCL)			X						X

7. STUDY OUTCOMES

7.1. Primary outcome

- Liver fat
  IHCL by ^{1}H-MRS measured at weeks 0 and 24
  [Time Frame: End value at week 24]

7.2. Secondary outcomes

- Whole body insulin sensitivity
  75g OGTT derived Matsuda whole body insulin sensitivity index (Matsuda ISI) at weeks 0 and 24
  [Time Frame: End value at week 24]

- Beta-cell function
  75g-OGTT derived insulin sensitivity index-2 (ISSI-2) measured at weeks 0 and 24
  [Time Frame: End value at week 24]

- Glucose tolerance – plasma glucose area under the curve (AUC)
  75g OGTT derived plasma glucose AUC measured at weeks 0 and 24
  [Time Frame: End value at week 24]

- Glucose tolerance – 2-hour plasma glucose (2h-PG)
  75g OGTT derived 2h-PG measured at weeks 0 and 24
  [Time Frame: End value at week 24]

7.3. Other Pre-specified outcomes

- Ectopic muscle fat
  IMCL in calf muscles by ^{1}H-MRS measured at weeks 0 and 24
  [Time Frame: End value at week 24]

- MetS criteria: waist circumference
  Waist circumference measured at weeks 0, 4 ,8, 12, 16, 20 and 24
  [Time Frame: End value at week 24]

- MetS criteria: FPG
  Fasting plasma glucose concentration measured at weeks 0 and 24

[Time Frame: End value at week 24]

- MetS criteria: Triglycerides
  Fasting serum triglycerides concentration measured at weeks 0 and 24
  [Time Frame: End value at week 24]

- MetS criteria: HDL-C
  Fasting serum HDL cholesterol concentration measured at weeks 0 and 24
  [Time Frame: End value at week 24]

- MetS criteria: SBP
  SBP measured at weeks 0, 4 ,8, 12, 16, 20 and 24
  [Time Frame: End value at week 24]

- MetS criteria: DBP
  DBP measured at weeks 0, 4 ,8, 12, 16, 20 and 24
  [Time Frame: End value at week 24]

- MetS Reversion
  Reversion to <3 of 5 IDF/NHLBI/AHA/WHF/IAAS/IASO MetS criteria measured at weeks 0 and 24
  [Time Frame: End value at week 24]

- Diabetes Incidence
  Incidence of diabetes (Diabetes Canada 2018 FPG and 2h-PG [75g-OGTT], or HbA1c criteria) measured at week 24
  [Time Frame: End value at week 24]

- Body weight
  Body weight measured at weeks 0, 4 ,8, 12, 16, 20 and 24
  [Time Frame: End value at week 24]

- Microbiome diversity
  Gut microbiome composition by 16S rRNA sequencing measured at weeks 0 and 24
  [Time Frame: End value at week 24]

- NAFL markers - ALT
  Serum ALT concentrations measured at weeks 0 and 24
  [Time Frame: End value at week 24]

- NAFL markers - AST
  Serum AST concentrations measured at weeks 0 and 24
  [Time Frame: End value at week 24]

- NAFL markers - GGT
  Serum GGT concentrations measured at weeks 0 and 24
  [Time Frame: End value at week 24]

- NAFL markers - ALP
  Serum ALP concentrations measured at weeks 0 and 24
  [Time Frame: End value at week 24]

- NAFL markers – FLI
  FLI measured at weeks 0 and 24
  [Time Frame: End value at week 24]

- Hepatic insulin resistance - HOMA-IR
  Homeostatic model assessment of insulin resistance (HOMA-IR) measured at weeks 0 and 24
  [Time Frame: End value at week 24]

- Uric acid
  Serum uric acid measured at weeks 0 and 24
  [Time Frame: End value at week 24]

- Established lipid targets - LDL-C
  Serum LDL-C measured at weeks 0 and 24
  [Time Frame: End value at week 24]

- Established lipid targets - non-HDL-C
  Serum non-HDL-C measured at weeks 0 and 24
  [Time Frame: End value at week 24]

- Established lipid targets - total Cholesterol
  Serum total Cholesterol measured at weeks 0 and 24
  [Time Frame: End value at week 24]

- Inflammation - hs-CRP
  Serum hs-CRP measured at weeks 0 and 24
  [Time Frame: End value at week 24]

- Kidney function/injury - creatinine
  Serum creatinine measured at weeks 0 and 24
  [Time Frame: End value at week 24]

- Kidney function/injury - eGFR
  eGFR measured at weeks 0 and 24
  [Time Frame: End value at week 24]

- Kidney function/injury - urinary albumin excretion rate (AER)
  Urinary AER measured from a 24 hour urine collection at weeks 0 and 24
  [Time Frame: End value at 24]

- Kidney function/injury - urinary albumin/creatinine ratio (ACR)
  Urinary ACR measured from a 24 hour urine collection at weeks 0 and 24
  [Time Frame: End value at 24]

- Diet quality
  The AHEI is comprised of 10 food components with each component scored on a 0 to 10 point scale and summed. Where 0 is the lowest and 110 is the highest total score. Assessments will be done at weeks 0 and 24. The HEI is comprised of 13 food components with each component scored a maximum of 5-10 point scale and summed. Where 0 is the lowest and 100 is the highest total score. Assessments will be done at weeks 0, 12 and 24.
  [Time Frame: End value at week 24]

- Appetite
  Appetite assessed by Control of Eating Questionnaire (CoEQ). The CoEQ comprises 21 items related to six sections: appetite, craving control, craving for sweet, craving for savoury, positive mood, perceived control over resisting a specific craved food. Items are assessed using 100-mm visual analogue scales, where 0 mm is lowest and 100 mm is highest. Assessments will be done at weeks 0, 4, 8, 12, 16, 20 and 24.
  [Time Frame: End value at week 24]

- Food craving control
  Food craving control assessed by Control of Eating Questionnaire (CoEQ). The CoEQ comprises 21 items related to six sections: appetite, craving control, craving for sweet, craving for savoury, positive mood, perceived control over resisting a specific craved food. Items are assessed using 100-mm visual analogue scales, where 0 mm is lowest and 100 mm is highest. Assessments will be done at weeks 0, 4, 8, 12, 16, 20 and 24.
  [Time Frame: End value at week 24]

- Food craving for savoury
  Food craving for savoury assessed by Control of Eating Questionnaire (CoEQ). The CoEQ comprises 21 items related to six sections: appetite, craving control, craving for sweet, craving for savoury, positive mood, perceived control over resisting a specific craved food. Items are assessed using 100-mm visual analogue scales, where 0 mm is lowest and 100 mm is highest. Assessments will be done at weeks 0, 4, 8, 12, 16, 20 and 24.
  [Time Frame: End value at week 24]

- Food craving for sweet
  Food craving for sweet assessed by Control of Eating Questionnaire (CoEQ). The CoEQ comprises 21 items related to six sections: appetite, craving control, craving for sweet, craving for savoury, positive mood, perceived control over resisting a specific craved food. Items are assessed using 100-mm visual analogue scales, where 0 mm is lowest and 100 mm is highest. Assessments will be done at weeks 0, 4, 8, 12, 16, 20 and 24.
  [Time Frame: End value at week 24]

- Perceived craving control over SSBs
  Perceived craving control over SSBs assessed by Control of Eating Questionnaire (CoEQ). The CoEQ comprises 21 items related to six sections: appetite, craving control, craving for sweet, craving for savoury, positive mood, perceived control over resisting a specific craved food. Items are assessed using 100-mm visual analogue scales, where 0 mm is lowest and 100 mm is highest. Assessments will be done at weeks 0, 4, 8, 12, 16, 20 and 24.
  [Time Frame: End value at week 24]

- Positive mood
  Positive mood assessed by Control of Eating Questionnaire (CoEQ). The CoEQ comprises 21 items related to six sections: appetite, craving control, craving for sweet, craving for savoury, positive mood, perceived control over resisting a specific craved food. Items are assessed using 100-mm visual analogue scales, where 0 mm is lowest and 100 mm is highest. Assessments will be done at weeks 0, 4, 8, 12, 16, 20 and 24.
  [Time Frame: End value at week 24]

- Adherence markers - Beverage logs
  Adherence by self-report beverage logs measured at weeks 0, 4, 8, 12, 16, 20 and 24
  [Time Frame: End value at week 24]

- Adherence biomarkers - Objective biomarker of soy milk
  Urinary isoflavonones excretion [UIE] measured at weeks 0, 12, and 24.
  [Time Frame: End value at week 24]

- Adherence biomarkers - Objective biomarkers of cow's milk
  Dairy-derived serum fatty acids [15:0, 17:0, conjugated linoleic acid, trans-palmitoleic acid] measured at weeks 0, 12, and 24.
  [Time Frame: End value at week 24]

- Adherence biomarkers - Objective biomarkers of SSBs (urinary fructose and sucrose)
  urinary fructose measured at weeks 0, 12, and 24.
  [Time Frame: End value at week 24]

8. PROCEDURES

8.1. Compliance assessment

To assess compliance, beverage logs will be checked at each visit. In addition, urinary analysis and blood analysis will be done (see adherence outcomes) at 3 time points: at the beginning (week 0), in the middle of the trial (week 12) and end of the trial (week 24).

8.2. Analytical techniques

8.2.1. Anthropometric and blood pressure analyses

Height will be measured with a wall-mounted stadiometer (Perspective Enterprises, Portage, MI, USA). Body weight will be assessed by beam scale. Waist circumference will be assessed using the Heart and Stroke Foundation methodology [64]. Blood pressure (BP) and resting heart rate will be measured. To collect this measure, participants will remain seated in a quiet, temperature-controlled room for at least 5 minutes to achieve resting heart rate and BP. Subsequently, BP will be measured oscillometrically using the OMRON Intellisense HEM-907 according to JNC VII criteria. BP will be measured in triplicate, with each measurement separated by one minute, and the average of the three measures will be taken [65].

8.2.2. Biochemical analyses

The fecal microbiome analysis and the objective biomarkers of the beverage assays will be performed at the Department of Nutritional Sciences, University of Toronto; plasma glucose, plasma insulin, liver enzymes, inflammation marker, uric acid, lipid analyses and kidney function analyses will be performed at the Banting and Best Diabetes Center (BBDC), University of Toronto.

8.2.2.1. Fecal microbiome analysis

DNA will be extracted from fecal samples and compositional analysis will be done by next generation sequencing. Primers will be used to target the appropriate regions of the *16S rRNA* gene for pair-end sequencing using Illumina MiSeq [66]. Sequencing data will be analyzed to assign Operational Taxonomic Units (OTUs) to determine their abundance. Data for selected taxa will be confirmed by qPCR [67]. Alpha and beta diversity indexes will be calculated. The metagenome will be inferred from compositional data *in silico* [68].

8.2.2.2. Plasma analyses

Plasma samples for glucose and insulin will be separated by centrifuge and the plasma immediately frozen at –80º C at the University of Toronto, in a locked room, in a locked building for analysis. The BBDC at the University of Toronto will perform analyses of the plasma glucose using the glucose oxidase

method [69] and plasma insulin using the Cobas Elecsys insulin assay (Roche Diagnostics GmbH, Mannheim, Germany), and of the serum liver enzymes (ALT, AST, GGT, ALP), inflammation marker (hs-CRP), uric acid, HbA1c and lipid analyses (Triglycerides, HDL-C, LDL-C, and total cholesterol). Plasma glucose and insulin curves will be plotted as the incremental change over time and iAUC will be calculated geometrically for each participant using the trapezoid method, ignoring areas below the fasting value [70]. Remaining samples will be stored at -80º C for future analysis of metabolomics. The Matsuda ISI_{OGTT} will be calculated using the 75g-OGTT derived plasma glucose (PG) and insulin (PI) values, according to the formula by Matsuda et al. [34]: 10 000 divided by the square root of ([FPGFPI] ([mean PG mean PI]), where PG is expressed in mg/dl (0.0551mmol/L) and PI in U/ml (6pmol/L). The early insulin secretion index ($\Delta PI_{30-0}/\Delta PG_{30-0}$) will be calculated as the change in PI from 0 minutes to 30 minutes divided by the change in PG over the same period [71].

8.2.2.3. Urinary analyses

Urine samples for albumin and creatinine will be frozen at –80º C at the University of Toronto, in a locked room, in a locked building for analysis. The BBDC at the University of Toronto will perform analyses of the urine albumin and creatine using the Jaffé alkaline picrate method[72].

8.2.2.4. Biomarkers of adherence

All objective biomarkers of adherence will be assessed at the Department of Nutritional Sciences, University of Toronto and will be conducted using stored serum samples at -80ºC. 2% soy milk intake adherence will be assessed by UIE and will be analyzed by liquid chromatography isotope-dilution tandem mass spectrometry (LCMSMS) [42]. 2% cow's milk adherence will be assessed by dairy-derived serum fatty acids, and will be analyzed by serum quantification of four fatty acids: 15:0, 17:0, CLA and TPA[43]. The fatty acids will be extracted as total lipids using the Folch et al. method [73] and they will be separated and quantified using modified methods as described by Abdelmagid et al.[74]. SSBs adherence will be assessed by urinary fructose and sucrose. Urinary fructose and sucrose will be measured by gas chromatography-mass spectrometry (GC-MS)[75].

8.2.3. Dietary Assessment

Diet quality will be measured with the AHEI using the Harvard Willett FFQ [40] and with the HEI using the online Diet ID [41] platform. We will be using the booklet 07 FFQ, a self-administered questionnaire to assess the intake of various nutrients. Nutrient intakes will be computed by assigning a daily frequency weight. Additionally, participants will be doing a digital assessment of their diets at three time points for the study (week 0, week 12 and week 24) using Diet ID. For this digital assessment, participants will be given a unique code identifier to log into the Diet ID web-based software via a custom study link that will be accessed by all participants for the required timepoints. Diet ID will prompt participants to view a series of food images, each representing unique diet patterns. After choosing the best match, participants then indicate diet preferences and enter biometric data (self-reported height, weight, age, sex, activity level).

8.2.4. Medical Imaging

For the ^{1}H-MRS we will use a 3T Siemens Magnetom Skyra scanner (Erlangen, Germany) with either an 18 channel body phased array coil or a dedicated peripheral phased array coil. Single voxel, 30mm cubed spectroscopy will be acquired for IHCL and IMCL using a STEAM (Stimulated Echo Acquisition Mode) sequence and the following acquisition parameters: TR 3000 ms, multiple TE values 12 ms, 24 ms, 36 ms, 48 ms, 72 ms, mixing time (TM) 10 ms, 10 averages, 1024 data points and receiver bandwidth 1200 Hz for an acquisition time under one minute. Participants will be scanned for 30 minutes. Participants with anxiety and/or claustrophobia, will be given the option to take oral medication to minimize the anxiety.

8.3. Data Integrity/Management

All paper copies of data forms will be stored under a double lock system to ensure confidentiality (locked cabinet in a locked office). Only PI, co-investigators and assigned study personnel will have access to the cabinet. The participant digital database will be securely stored centrally at SMH. The data management system enables anonymized and secure information storage, retrieval and sharing and will be organized and supported by the Li Ka Shing Knowledge Institute IT team of St. Michael's Hospital. All data forms will be stored under a double lock system to ensure confidentiality. Only the PI, co-investigators and assigned trial personnel will have access to the cabinet. The AHRC, St. Michael's Hospital, will be contracted to manage the digital database securely and ensure data integrity. Their data management system enables anonymization, secure information storage, retrieval, and sharing of data.

8.3.1. Personal Health Information

We will be collecting personal health information during this study. The personal health information will include name, phone number, email address, demographic information, health history, questionnaires and records, and research visit records. In order to keep participant data confidential, we will create a master linking log which will separate identifying data (name and contact information) from study data with a study ID. The master linking log will be kept in a password-protected file on a drive through St Michael's Hospital computer, only accessible to study staff and investigators. Any subject requesting access to their personal health information will be able to do so by requesting this through the study coordinator.

8.3.2. Trialfacts

Potential participants will see the advertisement for the study and their participation will be voluntary. If they choose to participate in the survey, they will be asked for personal information such as complete name, email address, and contact number. If potential participants decide to continue, they will be asked to respond to questions regarding the study eligibility criteria. At any point, the potential participant will have the opportunity to stop the survey before completing it, and any information used

up to that point will not be used. However, if the potential participant completes the survey, they will be giving their consent to us to collect their answers to the survey, including their contact information. While completing the survey they will have the option to join the mailing list to be contacted via email about future clinical trials for which they might be eligible. Trialfacts comply with the Privacy Act 1998 (Cth) (Privacy Act) and specifically the Australian Privacy Principles (APP). In addition, they comply with the HIPAA (Privacy Rule which is the primary protection regime under US law and applies to personal health information) and PHIPA (Personal Health Information Protection Act which is the Ontario's health privacy legislation). Trialfacts will delete the information from their records upon the potential participants' request or will de-identify personal information once it is no longer needed for a valid purpose or required to be kept by law.

8.3.3. Honeybee

Potential participants will see the advertisement for the study and their participation will be voluntary. If they choose to participate in the survey, before they start the survey they will be prompt to log in to their existing Honeybee account if they have one, to create a new Honeybee account or to skip the survey and contact the STEM trial team directly via email. They will be asked for personal information such as complete name, email address, and contact number. If potential participants decide to continue, they will be asked to respond to questions regarding the study eligibility criteria. At any point, the potential participant will have the opportunity to stop the survey before completing it, and any information used up to that point will not be used. However, if the potential participant completes the survey, they will be giving their consent to us to collect their answers to the survey, including their contact information. Participants with a Honeybee account will have the option to opt-in to receive Honeybee newsletters which relays other relevant clinical trial or health studies to them based on their profile information. Honeybee is an entirely HIPAA and PIPEDA (Personal Information Protection and Electronic Documents Act) compliant platform. Honeybee will delete the information from their records upon the potential participants' request or will de-identify personal information once it is no longer needed for a valid purpose (audits, to ensure data integrity) or required to be kept by law.

8.3.4. Diet ID

No personal identifying information will be shared with this company. Instead of names or email addresses, a study ID will be used. Data received and processed by Diet ID incudes gender, age, height, weight, activity level, and nutrient analysis. The data will be housed eternal to St. Michael's Hospital within the Diet ID app server, Aptible, which is a HIPAA compliant hosting service that runs on AWS (Amazon Web Service). The study team will be provided access to the admin dashboard and will be able to log in with administration credentials and view/export limited participant data. The complete data export can be exported by an assigned administration staff. Administration staff will have a username and password that includes 2-step verification. Study data will be kept in a password-protected file on a drive through St. Michael's Hospital computer, only accessible to study staff and investigators.

8.4. Subject Compensation

Once a participant is randomized, travel compensation will be provided. To compensate for time required for study visits, each participant will be compensated a total of $350, which will be divided into

3 payments: $100 at beginning of the trial (week 0), $100 in the middle of the trial (week 12) and $150 at completion of the trial (week 24). Transportation costs (i.e. parking or TTC tokens) will be reimbursed (up to $25 per visit) and breakfast will also be provided at each study visit. Any transportation costs incurred for additional visits for obtaining fecal and urine collection kits will be reimbursed as well.

8.5. Anticipated Challenges

8.5.1. Recruitment challenges

We require individuals who are regular SSB drinkers. In fact, soft drinks are the second largest source of added sugars for adults in Canada [76] and the per capita intake of SSBs in Canada is 0.9 with estimated 20 percent drinking more than 1 SSB serving per day [77], there is estimated 10 percent adult population that drinks more than 1 SSB serving per day. We plan to recruit from overweight and obese individuals who are known to have a higher SSB intake [78]. In addition, there are new challenges due to COVID-19 restrictions.

8.5.2. Compliance challenges

In dietary studies, compliance is usually a challenge. We will address this by having participants keep beverage logs during the trial. Beverages will be provided to the participants from the study site for the first week of each visit and shipped via Starhawk express for the three subsequent weeks. In addition, adherence to treatment will be reinforced with regular phone calls and/or emails to the participants.

9. Incidental findings and incident diabetes

An incidental finding is a finding concerning an individual research participant that has potential health or reproductive importance and is discovered in the course of conducting research but is beyond the aims of the study [79]. Any incidental findings (as defined above) obtained during the course of the main study from laboratory tests or ^{1}HMRS studies will be provided to the family physician for further investigation. Participants will be notified in reassuring terms and advised to follow-up with their family physician. If a participant does not have a family physician at the time of diagnosis, then specialist referrals will be made as necessary. If genes identified have clinical implications the advice of experts in that particular field will be sort to arrange appropriate counseling for the participant. The family physician's involvement would be sought if the participant consents to it. However, the chances of incidental findings are limited since only specific genes known to affect dietary response will be studied.

10. Banking of Samples

Blood samples (de-identified) will be stored at the University of Toronto (at the Medical Science Building) until all planned analyses have been undertaken-usually about 3 to 5 years. Longer-term banking storage for all samples will be 30 years after study completion to allow for use in future secondary and exploratory analyses in relation to the objectives of the current research project. At the

end of each storage period, samples will be destroyed by autoclaving. Presently the use and ultimate destruction of these samples will be the responsibility of the investigator, Dr. Sievenpiper.

11. Statistical Analysis

All statistical analyses will be conducted using STATA 14 (StataCorp, Texas, USA) or a suitable statistical software package on end values at week -24. The 3-arm design of the trial is intended to test the superiority of the active (2% soy milk) and reference (2% cow's milk) treatments compared with the control treatment (SSBs) and the noninferiority of the active treatment (2% soy milk) compared with the reference treatment (2% cow's milk).

The testing of the primary outcome will be done in a stepwise manner to decrease the familywise error rate. We will first undertake superiority testing of the active treatment (2% soy milk) and the reference treatment (2% cow's milk) compared with the control treatment (SSB). The primary analysis will be conducted according to the intention to treat (ITT) principle with inverse probability weighting (IPW) [https://www.bmj.com/content/370/bmj.m2215] to account for missing values. Data will be analyzed using ANCOVA models with significance set at a $p < 0.05$. Adjustments will made for for age, sex, NAFLD status, medication use, intervention dose (servings/day), and baseline level. Sensitivity analyses will include completers, per protocol, and ITT with multiple imputations (MI). If superiority is established in the primary analysis (confirming the "assay sensitivity"), then we will undertake non-inferiority testing of the active (2% soy milk) compared with the reference (2% cow's milk) treatment using per protocol analysis. If the upper bound of the 90% CI is less than the 1.5% non-inferiority margin [δ] by difference of means, then the active treatment (2% soy milk) will be considered non-inferior to the reference treatment (2% cow's milk). If the upper bound of the 90% CI of the difference of means is less than both the 1.5% non-inferiority margin [δ] and the 0 value (unity), then the active treatment (2% soy milk) will be considered superior to the reference treatment (2% cow's milk). The primary analysis for non-inferiority will be a per protocol analysis. Adjustments will made for for age, sex, NAFLD status, medication use, intervention dose (servings/day), and baseline level. Sensitivity analyses will include completers, ITT with IPW and ITT with MI.

Secondary, exploratory, and adherence outcomes will be assessed by superiority testing among the active treatment (2% soy milk), reference treatment (2% cow's milk), and control treatment (SSB). By reporting all estimated effects and confidence intervals with p-values, the necessary context is provided for a proper interpretation of the statistical evidence [https://www.tandfonline.com/doi/full/10.1080/00031305.2016.1154108]. If it is required for a future regulatory submission, then the secondary outcomes will be adjusted for false discovery. The approach will depend on the results of the primary outcome. If the superiority testing of the primary outcome is significant, then we will analyze the secondary outcomes using the Benjamini-Hochberg false discovery rate controlling method with a starting alpha of 0.05 to correct for false discovery [80].

The sample size was selected to allow sufficient power for this analytical approach. The primary analysis will be conducted according to the intention to treat (ITT) principle with IPW to account for any missing values. Data will be analyzed using ANCOVA models for continuous data (mean differences with 95% CIs) and logistic regression models for categorical data (odds ratio with 95% CI for MetS reversion and relative risk with 95% CI for diabetes incidence). Adjustments will made for for age, sex, NAFLD status, medication use, intervention dose (servings/day), and baseline level of the end value. Sensitivity analyses will include completers, per protocol analyses, and ITT with MI.

Prespecified subgroup analyses will be conducted using Chi-square tests by age, sex, NAFLD status, caffeinated beverage use (cola vs non-cola drinkers), intervention dose (servings/day), and baseline waist circumference, FPG, 2hPG, Triglycerides, HDL-C, BP. Continuous linear (dose response gradient) and non-linear (dose response threshold) dose-response analyses will be undertaken over the natural dose response range (3, 4, 5, or 6 servings per day) by multiple linear regression and piecewise regression analyses, respectively.

12. Significance of work

The proposed *Soy Treatment Evaluation on the progression of Metabolic Syndrome (STEM)* trial will strengthen the evidence-base for dietary recommendations that influence public health policy and programs and food procurement policies in public institutions, providing high-quality clinical evidence to support the use of soy as a “public health intervention” for addressing the epidemics of obesity and diabetes and overall metabolic health.

The 3-arm non-inferiority design provides the greatest opportunity for the success of the *STEM* trial. We hypothesize that the active 2% soy milk treatment will be superior to the negative SSBs control treatment. It is also expected to be superior to the reference 2% cow's milk treatment (the ‘standard of care’ from a public health perspective) based on previous head-to-head comparisons of soy milk with cow’s milk [7, 31, 32] and soy protein with dairy-derived casein [33]. To protect against the possibility of a lack of superiority, the pre-specification of a non-inferiority comparison with the reference 2% cow’s milk requires that it only has to be “as good as” the cow's milk to satisfy the research hypothesis. The present design also still retains the ability to test the superiority of the active 2% soy milk to the reference 2% cow's milk, as we have prespecified rules for testing superiority once non-inferiority criteria has been met. Either finding (non-inferiority or superiority) would represent an actionable outcome for soy, as it would position soy as a healthy alternative to cow’s milk for SSBs reduction policies and programs.

13. TIMELINE

Milestones	Dates	Timeline (years)
1. Research ethics/materials/data transfer approvals	Jun/21-Sep/21	4-Months
2. Rolling recruitment	Nov/21-Oct/22	12-Months
3. Run-in phase & intervention	Dec/21-Jun/23	19-Months
4. 1H-MRS/Blood/urine/fecal analyses	Jul/23-Dec/23	6-Months
5. Data analyses	Jan/24-Mar/24	3-Months
6. Final write-up	Apr/24-Jun/24	3-Months

14. Genetic Testing

This aspect of the study is not part of the main objectives of the study

14.1. Objective

To determine whether there are any genetic differences between individuals in their response to study beverages.

14.2. Background

There is considerable interest in the scientific community to study how genes may influence response to diet. In terms of taste our diets tend to be bland containing lower salt and also less sugar. The genes controlling these tastes may therefore determine intake [81, 82]. This is an example of why comprehensive genetic analysis, due to the implication of a growing number of genes of interest, would be helpful. We will, however limit our analyses to specific genes currently known to affect dietary response. Generally, the effect of genes on diet is very modest and usually requires significant numbers of people to detect the difference. It therefore has very limited clinical relevance. We may therefore pool data from across studies, including future studies (typically 30-180 participants per study). We hope to make our initial findings known to participants at the end of the study, especially if we discover specific groups for which certain types of dietary advice may prove advantageous. Genetic testing results are solely for use in this study and will not be shared with any third parties (i.e. insurance companies or the hospital).

14.3. Analyses

The DNA will be extracted from “buffy coat” of white blood cells obtained after plasma has been removed from citrated blood samples drawn at week 0 or any time point during the main study. Therefore, no additional blood samples will be required for this study. The genes of interest will include those related to the effectiveness of dietary change on blood glucose, cholesterol and associated measurements including blood pressure, taste preference, and other related genes which may influence response to diet.

REFERENCES

1. Anderson, T.J., et al., *2016 Canadian Cardiovascular Society Guidelines for the Management of Dyslipidemia for the Prevention of Cardiovascular Disease in the Adult.* Can J Cardiol, 2016. **32**(11): p. 1263-1282.
2. Diabetes Canada Clinical Practice Guidelines Expert, C., et al., *Nutrition Therapy.* Can J Diabetes, 2018. **42 Suppl 1**: p. S64-S79.
3. Wharton, S., et al., *Obesity in adults: a clinical practice guideline.* CMAJ, 2020. **192**(31): p. E875-E891.
4. Grundy, S.M., et al., *2018 AHA/ACC/AACVPR/AAPA/ABC/ACPM/ADA/AGS/APhA/ASPC/NLA/PCNA Guideline on the Management of Blood Cholesterol: A Report of the American College of Cardiology/American Heart Association Task Force on Clinical Practice Guidelines.* Circulation, 2019. **139**(25): p. e1082-e1143.
5. Stroes, E.S., et al., *Statin-associated muscle symptoms: impact on statin therapy-European Atherosclerosis Society Consensus Panel Statement on Assessment, Aetiology and Management.* Eur Heart J, 2015. **36**(17): p. 1012-22.
6. Catapano, A.L., et al., *2016 ESC/EAS Guidelines for the Management of Dyslipidaemias.* Rev Esp Cardiol (Engl Ed), 2017. **70**(2): p. 115.
7. Li, S.S., et al., *Effect of Plant Protein on Blood Lipids: A Systematic Review and Meta-Analysis of Randomized Controlled Trials.* J Am Heart Assoc, 2017. **6**(12).
8. Jenkins, D.J.A., et al., *Cumulative Meta-Analysis of the Soy Effect Over Time.* J Am Heart Assoc, 2019. **8**(13): p. e012458.
9. Blanco Mejia, S., et al., *A Meta-Analysis of 46 Studies Identified by the FDA Demonstrates that Soy Protein Decreases Circulating LDL and Total Cholesterol Concentrations in Adults.* J Nutr, 2019. **149**(6): p. 968-981.
10. Department of Health and Human Services(HHS). *Food Labeling: Health Claims; Soy Protein and Coronary Heart Disease* Available from: https://www.reginfo.gov/public/do/eAgendaViewRule?pubId=202010&RIN=0910-AH43.
11. The Centers for Disease Control. *The CDC Guide to Strategies for Reducing the Consumption of Sugar-Sweetened Beverages*. Available from: https://stacks.cdc.gov/view/cdc/51532.
12. *World Health Organization. Sugars Intake for Adults and Children: Guideline. Geneva, Switzerland: WHO; 2015*. Available from: https://www.who.int/publications/i/item/9789241549028.
13. *Scientific Report of the 2015 Dietary Guidelines Advisory Committee [Internet]. Washington (DC): Department of Health and Human Services and USDA. c2015 [cited 2016 Feb 16]*. Available from: https://health.gov/sites/default/files/2019-09/Scientific-Report-of-the-2015-Dietary-Guidelines-Advisory-Committee.pdf.

14. Scientific Advisory Committee on Nutrition. *Carbohydrates and Health Report*. Available from: https://assets.publishing.service.gov.uk/government/uploads/system/uploads/attachment_data/file/445503/SACN_Carbohydrates_and_Health.pdf.
15. Government of Canada. *Health Canada. Canada's Food Guide: Canada's Dietary Guidelines for Health Professionals and Policy Makers*. Available from: https://food-guide.canada.ca/en/guidelines/.
16. Diabetes Canada. *Sugar & diabetes: Position statement*. Available from: https://www.diabetes.ca/advocacy---policies/our-policy-positions/sugar---diabetes#:~:text=Position%20statement,on%20a%202000%2Dcalorie%20diet.
17. International Diabetes Federation. *IDF Europe Position Paper on Added Sugar*. Available from: https://www.eu-patient.eu/globalassets/library/publications/added-sugar-final_idf-europe-position.pdf.
18. Johnson, R.K., et al., *Dietary sugars intake and cardiovascular health: a scientific statement from the American Heart Association.* Circulation, 2009. **120**(11): p. 1011-20.
19. Heart and Stroke Foundation of Canada. *Reduce sugar*. Available from: https://www.heartandstroke.ca/healthy-living/healthy-eating/reduce-sugar.
20. Soares, A.A., *Putting taxes into the diet equation.* Bull World Health Organ, 2016. **94**(4): p. 239-40.
21. Bascunan, J. and C. Cuadrado, *Effectiveness of sugar-sweetened beverages taxes to reduce obesity: evidence brief for policy*. Medwave, 2017. **17**(8): p. e7054.
22. UK Government. *Soft Drinks Industry Levy Comes Into Effect*. Available from: https://www.gov.uk/government/news/soft-drinks-industry-levy-comes-into-effect.
23. Government of Quebec. *Going the healthy route at school Pamphlet 2 — Healthy Vending Machines*. Available from: http://www.education.gouv.qc.ca/fileadmin/site_web/documents/dpse/adaptation_serv_compl/virage-sante_fiche2_machines-distributrices_AN.pdf.
24. National Academies of Sciences, E., et al., in *Strategies to Limit Sugar-Sweetened Beverage Consumption in Young Children: Proceedings of a Workshop*. 2017, National Academies Press (US). Copyright 2017 by the National Academy of Sciences. All rights reserved.: Washington (DC).
25. Center for Science in the Public Interest, *Encouraging Healthier Choices in Hospitals.*
26. NHS. *Sugar: the facts-Eat well*. Available from: https://www.nhs.uk/live-well/eat-well/how-does-sugar-in-our-diet-affect-our-health/.
27. EU Science Hub. *Sugars and Sweeteners*. Available from: https://ec.europa.eu/jrc/en/health-knowledge-gateway/promotion-prevention/nutrition/sugars-sweeteners.
28. Pan, A., et al., *Changes in water and beverage intake and long-term weight changes: results from three prospective cohort studies.* Int J Obes (Lond), 2013. **37**(10): p. 1378-85.
29. Pan, A., et al., *Plain-water intake and risk of type 2 diabetes in young and middle-aged women.* Am J Clin Nutr, 2012. **95**(6): p. 1454-60.
30. Maersk, M., et al., *Sucrose-sweetened beverages increase fat storage in the liver, muscle, and visceral fat depot: a 6-mo randomized intervention study.* Am J Clin Nutr, 2012. **95**(2): p. 283-9.

31. Maleki, Z., et al., *Effect of soy milk consumption on glycemic status, blood pressure, fibrinogen and malondialdehyde in patients with non-alcoholic fatty liver disease: a randomized controlled trial.* Complement Ther Med, 2019. **44**: p. 44-50.
32. Eslami, O., et al., *Effect of Soy Milk on Metabolic Status of Patients with Nonalcoholic Fatty Liver Disease: A Randomized Clinical Trial.* J Am Coll Nutr, 2019. **38**(1): p. 51-58.
33. Kohno, M., et al., *Decreases in serum triacylglycerol and visceral fat mediated by dietary soybean beta-conglycinin.* J Atheroscler Thromb, 2006. **13**(5): p. 247-55.
34. Matsuda, M. and R.A. DeFronzo, *Insulin sensitivity indices obtained from oral glucose tolerance testing: comparison with the euglycemic insulin clamp.* Diabetes Care, 1999. **22**(9): p. 1462-70.
35. Retnakaran, R., et al., *Hyperbolic relationship between insulin secretion and sensitivity on oral glucose tolerance test.* Obesity (Silver Spring), 2008. **16**(8): p. 1901-7.
36. Retnakaran, R., et al., *Evaluation of proposed oral disposition index measures in relation to the actual disposition index.* Diabet Med, 2009. **26**(12): p. 1198-203.
37. Alberti, K.G., et al., *Harmonizing the metabolic syndrome: a joint interim statement of the International Diabetes Federation Task Force on Epidemiology and Prevention; National Heart, Lung, and Blood Institute; American Heart Association; World Heart Federation; International Atherosclerosis Society; and International Association for the Study of Obesity.* Circulation, 2009. **120**(16): p. 1640-5.
38. Punthakee, Z., R. Goldenberg, and P. Katz, *Definition, Classification and Diagnosis of Diabetes, Prediabetes and Metabolic Syndrome.* Can J Diabetes, 2018. **42 Suppl 1**: p. S10-s15.
39. Bedogni, G., et al., *The Fatty Liver Index: a simple and accurate predictor of hepatic steatosis in the general population.* BMC Gastroenterol, 2006. **6**: p. 33.
40. Ley, S.H., et al., *Changes in Overall Diet Quality and Subsequent Type 2 Diabetes Risk: Three U.S. Prospective Cohorts.* Diabetes Care, 2016. **39**(11): p. 2011-2018.
41. Katz, D.L., et al., *Dietary assessment can be based on pattern recognition rather than recall.* Med Hypotheses, 2020. **140**: p. 109644.
42. Morimoto, Y., et al., *Urinary isoflavonoid excretion as a biomarker of dietary soy intake during two randomized soy trials.* Asia Pac J Clin Nutr, 2014. **23**(2): p. 205-9.
43. Santaren, I.D., et al., *The Distribution of Fatty Acid Biomarkers of Dairy Intake across Serum Lipid Fractions: The Prospective Metabolism and Islet Cell Evaluation (PROMISE) Cohort.* Lipids, 2019. **54**(10): p. 617-627.
44. Piaggio, G., et al., *Reporting of noninferiority and equivalence randomized trials: extension of the CONSORT 2010 statement.* JAMA, 2012. **308**(24): p. 2594-604.
45. U.S. Department of Health and Human Services Food and Drug Administration Center for Drug Evaluation and Research (CDER), *Guidance for Industry Developing Products for Weight Management [DRAFT GUIDANCE].*
46. Evidence Analysis Library Division Center for Nutrition Policy and Promotion. *A Series of Systematic Reviews on the Relationship Between Dietary Patterns and Health Outcomes*. Available from: https://nesr.usda.gov/sites/default/files/2019-06/DietaryPatternsReport-FullFinal2.pdf.
47. U.S. Department of Agriculture and U.S. Department of Health and Human Services. *Dietary Guidelines for Americans, 2020-2025*. December 2020; 9th Edition:[Available from: https://www.dietaryguidelines.gov/.

48. Monteiro, C.A., et al. *Ultra-processed foods, diet quality, and health using the NOVA classification system*. 2019.
49. Crimarco, A., et al., *A randomized crossover trial on the effect of plant-based compared with animal-based meat on trimethylamine-N-oxide and cardiovascular disease risk factors in generally healthy adults: Study With Appetizing Plantfood-Meat Eating Alternative Trial (SWAP-MEAT).* Am J Clin Nutr, 2020. **112**(5): p. 1188-1199.
50. Pan American Health Organization. *Ultra-processed food and drink products in Latin America: Trends, impact on obesity, policy implications*. 2015; Available from: https://iris.paho.org/bitstream/handle/10665.2/7699/9789275118641_eng.pdf?sequence=5&isAllowed=y&ua=1.
51. Sun, W., S. Grosser, and Y. Tsong, *Ratio of means vs. difference of means as measures of superiority, noninferiority, and average bioequivalence.* J Biopharm Stat, 2017. **27**(2): p. 338-355.
52. Lazo, M., et al., *Effect of a 12-month intensive lifestyle intervention on hepatic steatosis in adults with type 2 diabetes.* Diabetes Care, 2010. **33**(10): p. 2156-63.
53. Rosqvist, F., et al., *Overeating Saturated Fat Promotes Fatty Liver and Ceramides Compared With Polyunsaturated Fat: A Randomized Trial.* J Clin Endocrinol Metab, 2019. **104**(12): p. 6207-6219.
54. Sobrecases, H., et al., *Effects of short-term overfeeding with fructose, fat and fructose plus fat on plasma and hepatic lipids in healthy men.* Diabetes Metab, 2010. **36**(3): p. 244-6.
55. Cowin, G.J., et al., *Magnetic resonance imaging and spectroscopy for monitoring liver steatosis.* J Magn Reson Imaging, 2008. **28**(4): p. 937-45.
56. Bortolotti, M., et al., *High protein intake reduces intrahepatocellular lipid deposition in humans.* Am J Clin Nutr, 2009. **90**(4): p. 1002-10.
57. Parry, S.A., et al., *Intrahepatic Fat and Postprandial Glycemia Increase After Consumption of a Diet Enriched in Saturated Fat Compared With Free Sugars.* Diabetes Care, 2020. **43**(5): p. 1134-1141.
58. U.S. Department of Health and Human Services, et al. *Non-Inferiority Clinical Trials to Establish Effectiveness, Guidance for Industry*. Available from: https://www.fda.gov/media/78504/download.
59. Committee for Medicinal Products for Human Use (CHMP). *Guideline on the choice of the non-inferiority margin*. Available from: https://www.ema.europa.eu/en/documents/scientific-guideline/guideline-choice-non-inferiority-margin_en.pdf.
60. Althunian, T.A., et al., *Defining the noninferiority margin and analysing noninferiority: An overview.* Br J Clin Pharmacol, 2017. **83**(8): p. 1636-1642.
61. Messina, M., et al., *Neither soyfoods nor isoflavones warrant classification as endocrine disruptors: a technical review of the observational and clinical data.* Crit Rev Food Sci Nutr, 2021: p. 1-57.
62. EFSA Panel on Food Additives and Nutrient Sources added to Food (ANS) *Risk assessment for peri- and post-menopausal women taking food supplements containing isolated isoflavones*. 2015. **13**.
63. *Diabetes mellitus. Report of a WHO Study Group.* World Health Organ Tech Rep Ser, 1985. **727**: p. 1-113.

64. Heart and Stroke Foundation of Canada. *Healthy weight and waist*. Available from: https://www.heartandstroke.ca/healthy-living/healthy-weight/healthy-weight-and-waist.
65. Blom, D.J., et al., *A 52-week placebo-controlled trial of evolocumab in hyperlipidemia.* N Engl J Med, 2014. **370**(19): p. 1809-19.
66. Caporaso, J.G., et al., *Ultra-high-throughput microbial community analysis on the Illumina HiSeq and MiSeq platforms.* ISME J, 2012. **6**(8): p. 1621-4.
67. Mouzaki, M., et al., *Intestinal microbiota in patients with nonalcoholic fatty liver disease.* Hepatology, 2013. **58**(1): p. 120-7.
68. Langille, M.G., et al., *Predictive functional profiling of microbial communities using 16S rRNA marker gene sequences.* Nat Biotechnol, 2013. **31**(9): p. 814-21.
69. Kadish, A.H. and D.A. Hall, *A new method for the continuous monitoring of blood glucose by measurement of dissolved oxygen.* Clin Chem, 1965. **11**(9): p. 869-75.
70. Wolever, T.M., et al., *The glycemic index: methodology and clinical implications.* Am J Clin Nutr, 1991. **54**(5): p. 846-54.
71. Phillips, D.I., et al., *Understanding oral glucose tolerance: comparison of glucose or insulin measurements during the oral glucose tolerance test with specific measurements of insulin resistance and insulin secretion.* Diabet Med, 1994. **11**(3): p. 286-92.
72. Junge, W., et al., *Determination of reference intervals for serum creatinine, creatinine excretion and creatinine clearance with an enzymatic and a modified Jaffe method.* Clin Chim Acta, 2004. **344**(1-2): p. 137-48.
73. Folch, J., M. Lees, and G.H. Sloane Stanley, *A simple method for the isolation and purification of total lipides from animal tissues.* J Biol Chem, 1957. **226**(1): p. 497-509.
74. Abdelmagid, S.A., et al., *Plasma concentration of cis9trans11 CLA in males and females is influenced by SCD1 genetic variations and hormonal contraceptives: a cross-sectional study.* Nutr Metab (Lond), 2013. **10**: p. 50.
75. Theytaz, F., et al., *Metabolic fate of fructose ingested with and without glucose in a mixed meal.* Nutrients, 2014. **6**(7): p. 2632-49.
76. Brisbois, T.D., et al., *Estimated intakes and sources of total and added sugars in the Canadian diet.* Nutrients, 2014. **6**(5): p. 1899-912.
77. Singh, G.M., et al., *Estimated Global, Regional, and National Disease Burdens Related to Sugar-Sweetened Beverage Consumption in 2010.* Circulation, 2015. **132**(8): p. 639-66.
78. Bleich, S.N., et al., *Increasing consumption of sugar-sweetened beverages among US adults: 1988-1994 to 1999-2004.* Am J Clin Nutr, 2009. **89**(1): p. 372-81.
79. Wolf, S.M., et al., *Managing incidental findings in human subjects research: analysis and recommendations.* J Law Med Ethics, 2008. **36**(2): p. 219-48, 211.
80. U.S. Food & Drug Administration. *Multiple Endpoints in Clinical Trials Guidance for Industry*. Available from: https://www.fda.gov/regulatory-information/search-fda-guidance-documents/multiple-endpoints-clinical-trials-guidance-industry.
81. Dias, A.G., et al., *Genetic variation in putative salt taste receptors and salt taste perception in humans.* Chem Senses, 2013. **38**(2): p. 137-45.
82. Eny, K.M., et al., *Genetic variant in the glucose transporter type 2 is associated with higher intakes of sugars in two distinct populations.* Physiol Genomics, 2008. **33**(3): p. 355-60.
